# A mechanistic investigation of the photoinduced, copper-mediated cross-coupling of an aryl thiol with an aryl halide†

**DOI:** 10.1039/c5sc04709a

**Published:** 2016-02-24

**Authors:** Miles W. Johnson, Kareem I. Hannoun, Yichen Tan, Gregory C. Fu, Jonas C. Peters

**Affiliations:** a Division of Chemistry and Chemical Engineering, California Institute of Technology Pasadena, CA 91125 USA gcfu@caltech.edu jpeters@caltech.edu

## Abstract

Photoinduced, copper-catalyzed cross-coupling can offer a complementary approach to thermal (non-photoinduced) methods for generating C–X (X = C, N, O, S, *etc.*) bonds. In this report, we describe the first detailed mechanistic investigation of one of the processes that we have developed, specifically, the (stoichiometric) coupling of a copper–thiolate with an aryl iodide. In particular, we focus on the chemistry of a discrete [Cu^I^(SAr)_2_]^−^ complex (Ar = 2,6-dimethylphenyl), applying a range of techniques, including ESI-MS, cyclic voltammetry, transient luminescence spectroscopy, optical spectroscopy, DFT calculations, Stern–Volmer analysis, EPR spectroscopy, actinometry, and reactivity studies. The available data are consistent with the viability of a pathway in which photoexcited [Cu^I^(SAr)_2_]^−^* serves as an electron donor to an aryl iodide to afford an aryl radical, which then reacts in cage with the newly generated copper(ii)–thiolate to furnish the cross-coupling product in a non-chain process.

## Abstract 2

A mechanistic study establishes the viability of a copper-mediated pathway for photoinduced C–S cross-coupling involving single electron transfer and in-cage radical recombination.

## Graphical abstract

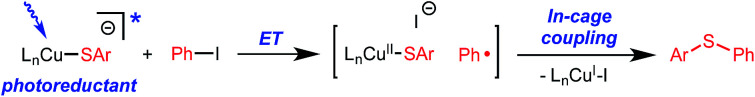

## Introduction

The utility of cross-coupling chemistry has continued to expand at a rapid rate as novel or underexplored reaction pathways are exploited to achieve important new families of bond constructions.^1^ We have recently reported that, in the presence of light and a simple copper catalyst, coupling reactions of a variety of nucleophiles (nitrogen, sulfur, oxygen, and carbon) with aryl or alkyl electrophiles can be accomplished under mild conditions (−40 to 30 °C; eqn (1)).^2–4^1
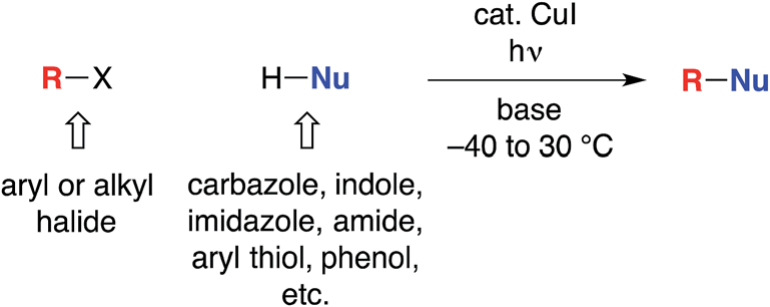


**Fig. 1:**
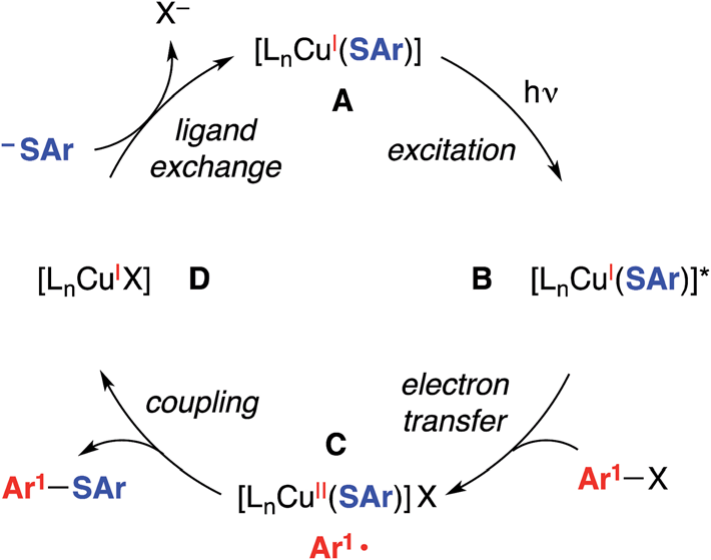
Outline of a possible catalytic cycle for photoinduced, copper-catalyzed cross-coupling: coupling of an aryl radical with a copper(ii)–thiolate as a key step.^5,11^

We have suggested the outline of a possible pathway for these processes (Fig. 1, illustrated for C–S coupling),^5^ recognizing that the course of the cross-coupling is likely to vary with different coupling partners and reaction conditions. We have been interested in mechanistic similarities and dichotomies with photoredox catalysis, a mode of reactivity that has been the focus of great interest in recent years.^6^ For example, we have hypothesized that, distinct from a classical photoredox catalyst wherein a particular metal complex serves exclusively as an electron donor/acceptor, in our processes the copper complex may play a role both in electron transfer and in the key bond-forming step (*e.g.*, C–S bond construction in Fig. 1).^2*a*–*d*,3,7^ Furthermore, the mechanism depicted in Fig. 1 is not a radical-chain process; although non-chain pathways have frequently been invoked in earlier studies of photoredox catalysis,^8^ Yoon has recently concluded that, for three representative and mechanistically distinct transformations, the photoredox catalyst serves to initiate a chain reaction.^9^ In this report, we describe our first study focused primarily on the mechanism of a photoinduced, copper-mediated cross-coupling, specifically, an investigation of the stoichiometric coupling of an aryl iodide with a copper–thiolate (eqn (2)).^10^2
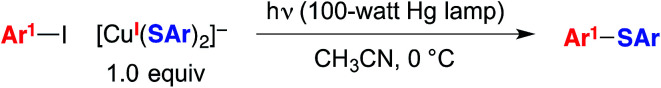


## Results and discussion

### Background

The photoinduced coupling of aryl thiols with aryl halides in liquid ammonia, in the absence of a catalyst, through an S_RN_1 mechanism is well-established through the work of Bunnett.^12–14^ In our initial report, we observed that a model photoinduced cross-coupling proceeds significantly more rapidly in the presence of a copper catalyst than in its absence (eqn (3)).^2*b*^3
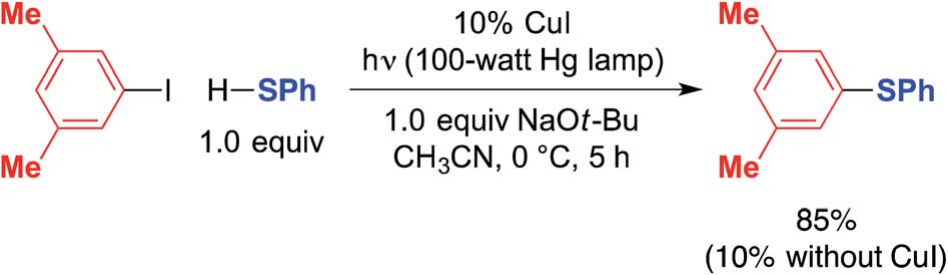


Under our reported conditions, the reaction mixture is heterogeneous, with a substantial portion of the NaSPh present as a solid. We have determined that, when the same partners are coupled at much lower concentration in a homogeneous solution, the rates of product formation can be similar in the presence and in the absence of CuI.^15^ Thus, a copper-mediated pathway and a copper-free pathway for C–S bond formation are possible, and which one is dominant can depend on the relative concentration in solution of sodium *versus* copper thiolates (the latter are generally more soluble in CH_3_CN). In the present investigation, we seek to gain insight into the copper-mediated pathway.

In the mechanistic framework that we have previously described (Fig. 1), irradiation of a copper(i)–thiolate complex (A) leads to a photoexcited state (B). Electron transfer from B to the aryl halide furnishes a copper(ii)–thiolate complex (C) and an aryl radical. Radical recombination then forms the C–S bond of the thioether, either directly^16^ or through a copper(iii) intermediate, and a copper(i)–halide complex (D). Displacement of the halide of complex D by thiolate then regenerates copper(i)–thiolate complex A.

**Fig. 2:**
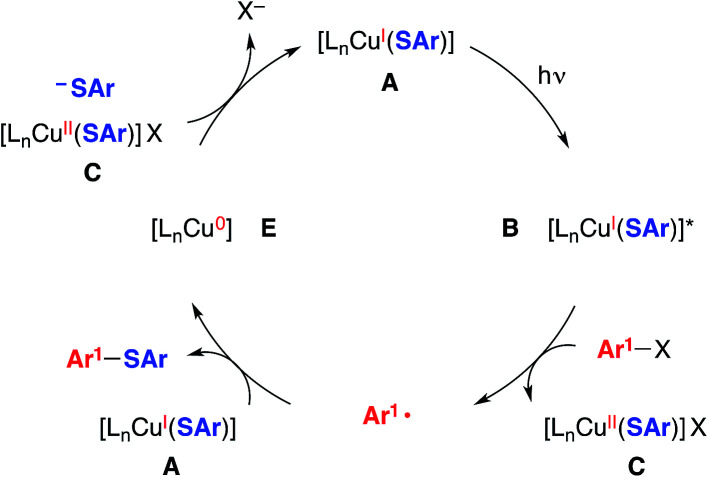
An alternative mechanism: coupling of an aryl radical with a copper(i)–thiolate as a key step.^5^

**Fig. 3:**
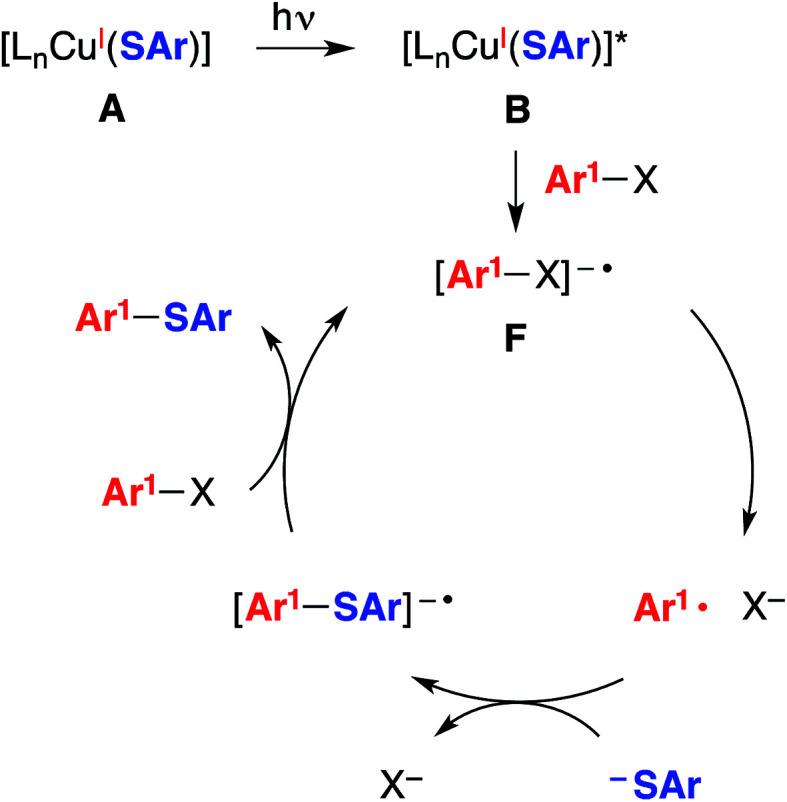
An alternative mechanism: S_RN_1 (radical chain process).^5^

**Fig. 4:**
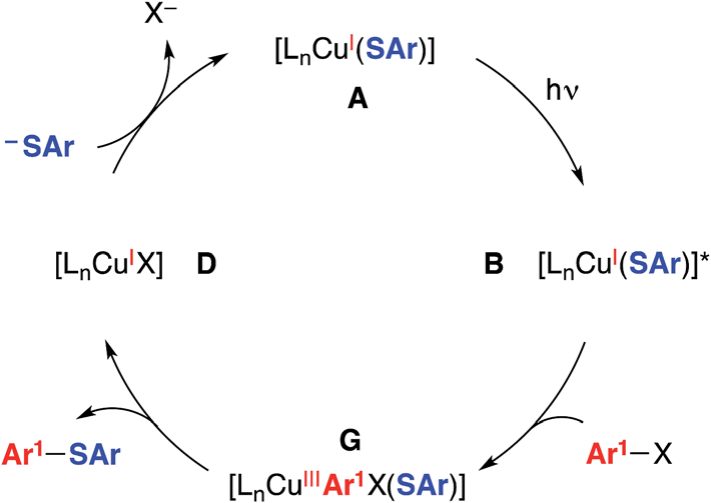
An alternative mechanism: concerted oxidative addition.^5,20^

We have also considered a variety of other mechanisms, including the three illustrated in Fig. 2–4. The pathway depicted in Fig. 2 produces an aryl radical and a copper(ii)–thiolate (C) through the same initial steps as in Fig. 1. Next, the aryl radical reacts with a copper(i)–thiolate (A), rather than a copper(ii)–thiolate (C; Fig. 1), to form the thioether, as well as copper(0) (E).^17^ Comproportionation of copper(0) with copper(ii)–thiolate C could regenerate copper(i)–thiolate A.

Another mechanism under consideration largely follows the S_RN_1 pathway for copper-free C–S coupling reactions,^12^ the difference being that a photoexcited copper(i)–thiolate (B), rather than a photoexcited copper-free thiolate, serves as the initiating electron donor to the aryl halide, thereby generating a radical anion (F) that can participate in a chain reaction to form the thioether (Fig. 3).^18^

We have also considered mechanisms that do not involve an organic radical as an intermediate. For example, in the pathway depicted in Fig. 4, photoexcited complex B reacts with the aryl halide to cleave the C–X bond in a concerted process without the intermediacy of an aryl radical.^19^ Reductive elimination of the resulting copper(iii) complex (G) leads to the thioether product (Ar^1^–SAr) and copper(i)–halide adduct D. Ligand exchange then completes the catalytic cycle by regenerating copper(i)–thiolate A.

### Previously reported mechanistic observations^2*b*^

In our original report, we described cyclization/stereochemistry data (eqn (4)) that are more readily accommodated by a radical/electron-transfer pathway (Fig. 1–3) than by a concerted pathway (Fig. 4) for C–X bond cleavage. Furthermore, in a relative-reactivity study (eqn (5)), we determined that the aryl halide that is more easily reduced *via* electron transfer (4-chlorobenzonitrile; −2.03 V *vs.* SCE in DMF^21^) is more reactive than the one with the weaker C–X bond (1-bromonaphthalene; −2.17 V *vs.* SCE in DMF^21^); this contrasts with thermal (non-photoinduced) copper-catalyzed *S*-arylation, wherein essentially exclusive coupling of the aryl bromide is proposed to result from concerted oxidative addition.^19,22^4
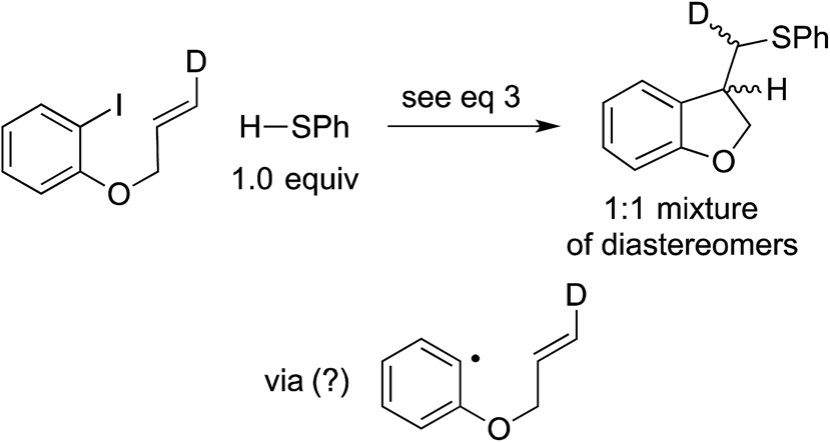
5
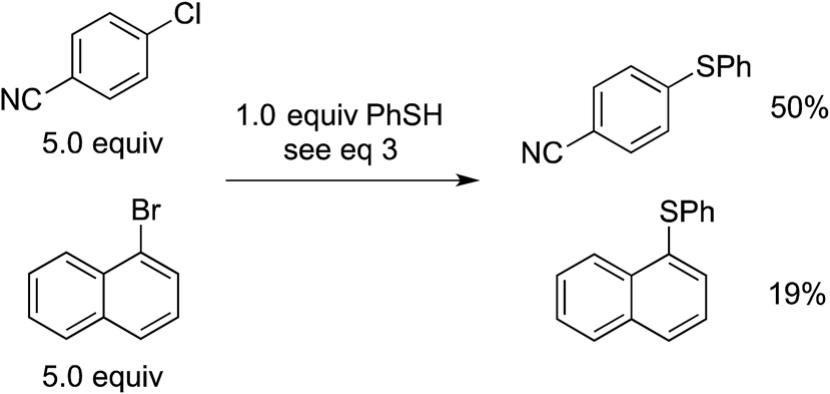


Our efforts to isolate a mononuclear [Cu^I^(SPh)_2_]^−^ complex (*e.g.*, A in Fig. 1–4), which we had detected in an ESI-MS study of a C–S coupling reaction, led instead to a copper(i)–thiolate cluster, [Cu^I^_5_(SPh)_7_][Na(12-crown-4)_2_]_2_. This cluster did, however, serve as a suitable stoichiometric coupling partner with an aryl iodide, as well as an effective (pre)catalyst for a photoinduced C–S cross-coupling.

Although these observations are consistent with our initial working hypothesis for the mechanism of photoinduced, copper-catalyzed C–S cross-couplings (Fig. 1), we concluded that a more detailed investigation was warranted.

### Synthesis and characterization of a monomeric copper(i)–thiolate model complex

**Fig. 5:**
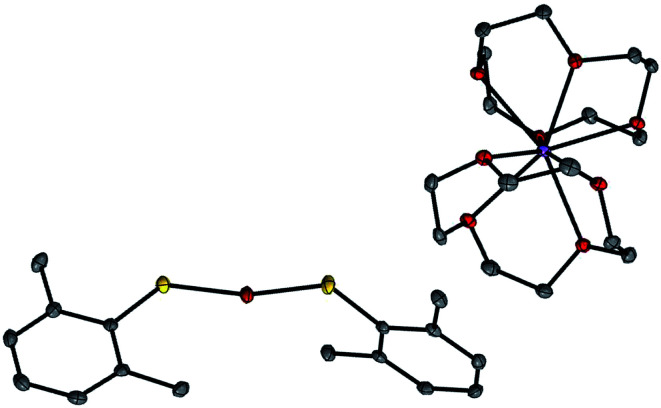
X-ray crystal structure of [Cu^I^(SAr)_2_][Na(12-crown-4)_2_] (1). Ellipsoids are shown at 50% probability, and hydrogen atoms have been omitted for clarity. Selected bond lengths and bond angle: Cu–S = 2.1477(5) Å and 2.1499(5) Å; S–Cu–S, 166.82(2)°.

A copper(i)–thiolate (A) is the starting point in each of the pathways illustrated in Fig. 1–4. For ease of analysis in the present investigation, we sought a model system of simple speciation (monomeric). As demonstrated by Tshuva, the use of a hindered arylthiolate (2,6-dimethylthiophenolate; SAr, Ar = 2,6-dimethylphenyl) can avoid the formation of a cluster;^23^ furthermore, we had reported in our initial study that this arylthiolate serves as a suitable coupling partner in photoinduced C–S cross-couplings.^2*b*^ Reaction of mesitylcopper(i), 2,6-dimethylthiophenol, and NaO*t*-Bu in CH_3_CN, followed by the addition of 12-crown-4, provided the desired sodium salt, [Cu^I^(SAr)_2_][Na(12-crown-4)_2_] (1; “[Cu^I^(SAr)_2_]Na”; eqn (6) and Fig. 5).6
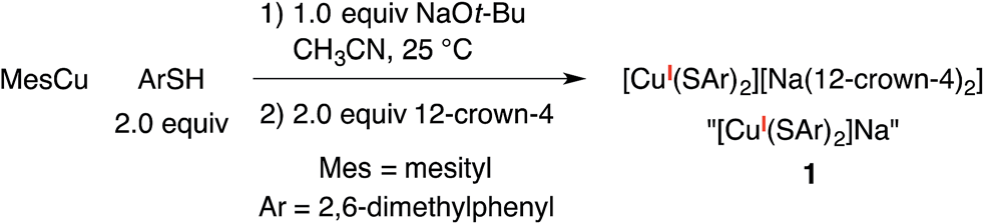


Our available data are consistent with the suggestion that this copper(i)–thiolate is a monomer in solution, as in the solid state. On the basis of diffusion-ordered NMR spectroscopy (DOSY), we estimate the hydrodynamic radii of the anion and the cation to be 4.2 and 4.4 Å, respectively, which are comparable to the corresponding computed radii of 4.5 and 4.8 Å. Furthermore, the molar conductivity for complex 1 in acetonitrile, 128.5 S cm^2^ mol^−1^, falls within the range (120–160 S cm^2^ mol^−1^) for other coordination compounds that are 1 : 1 electrolytes.^24^

NaSAr (Ar = 2,6-dimethylphenyl) is significantly more soluble in CH_3_CN than is NaSPh; consequently, for the photoinduced coupling of NaSAr with Ph–I under our standard conditions, the rates of reaction in the absence and in the presence of CuI are similar, in contrast to our observations with NaSPh (eqn (3)). Nevertheless, we have determined that [Cu^I^(SAr)_2_]Na (1) couples at 0 °C with Ph–I in 56% yield, thereby substantiating the viability of photoinduced copper-mediated *S*-arylation with this model complex (eqn (7)).7
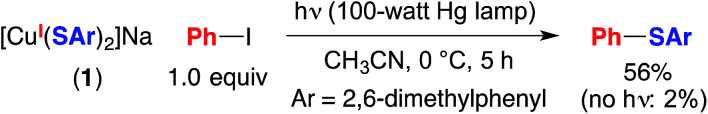


In an ESI-MS study of the coupling of ArSH with Ph–I under our standard copper-catalyzed cross-coupling conditions (eqn (3)), we have detected an anion with a molecular weight of 337.2, which corresponds to that of [Cu^I^(SAr)_2_]^−^; under these conditions, we do not observe [Cu^I^(SAr)_3_]^2−^, despite the large excess of thiolate relative to copper. Furthermore, ^1^H NMR and optical absorption spectra of complex 1 in the presence of excess thiolate, as well as DFT calculations,^25^ indicate that formation of [Cu^I^(SAr)_3_]^2−^ is unfavorable. Collectively, our data suggest that complex 1 exists as a two-coordinate monomer in solution, even in the presence of excess thiolate.

### Electrochemistry

**Fig. 6:**
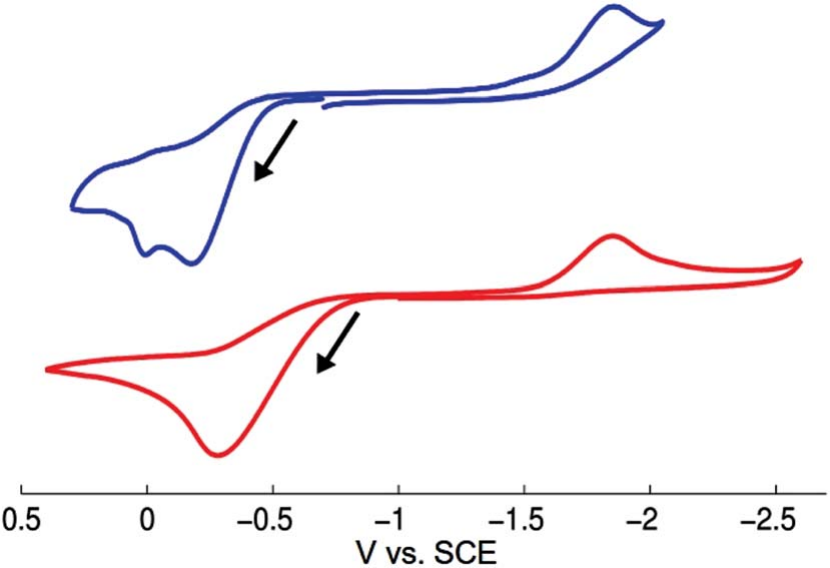
Cyclic voltammograms of [Cu^I^(SAr)_2_]Na (1; top) and of NaSAr (bottom). Conditions: scan rate: 100 mV s^−1^; supporting electrolyte: 0.08 M [(*n*-Bu)_4_N][B(C_6_F_5_)_4_]; working electrode: glassy carbon; reference electrode: Ag/AgNO_3_ (0.1 mM)/acetonitrile; auxiliary electrode: platinum wire; temperature: 25 °C.

We have examined through electrochemistry the redox behavior of [Cu^I^(SAr)_2_]Na (1) and of NaSAr (Fig. 6). The cyclic voltammogram of complex 1 shows an irreversible oxidative feature at *E*_p_ = −0.18 V *vs.* SCE that is also irreversible at −20 °C and at scan rates up to 1.5 V s^−1^ at 25 °C. Following oxidation of 1, an irreversible feature is observed at −1.85 V *vs.* SCE, which corresponds to the reduction of bis(2,6-dimethylphenyl) disulfide (ArS–SAr), presumably formed from complex 1 upon electrochemical oxidation (oxidation of 1 with [FeCp_2_][PF_6_] also leads to the formation of ArS–SAr).

To gain insight into whether free thiolate, generated either by simple ligand dissociation or through irradiation, might play a significant role in stoichiometric reactions of complex 1, we have monitored by cyclic voltammetry a solution of 1 (0.020 M) and [(*n*-Bu)_4_N][B(C_6_F_5_)_4_] as electrolyte in acetonitrile. The cyclic voltammogram is unchanged over 15 minutes of irradiation with a Hg lamp, suggesting that irradiation of complex 1 does not lead to the release of a detectable amount of free thiolate.

### Photophysical study of [Cu^I^(SAr)_2_]Na (1)

**Fig. 7:**
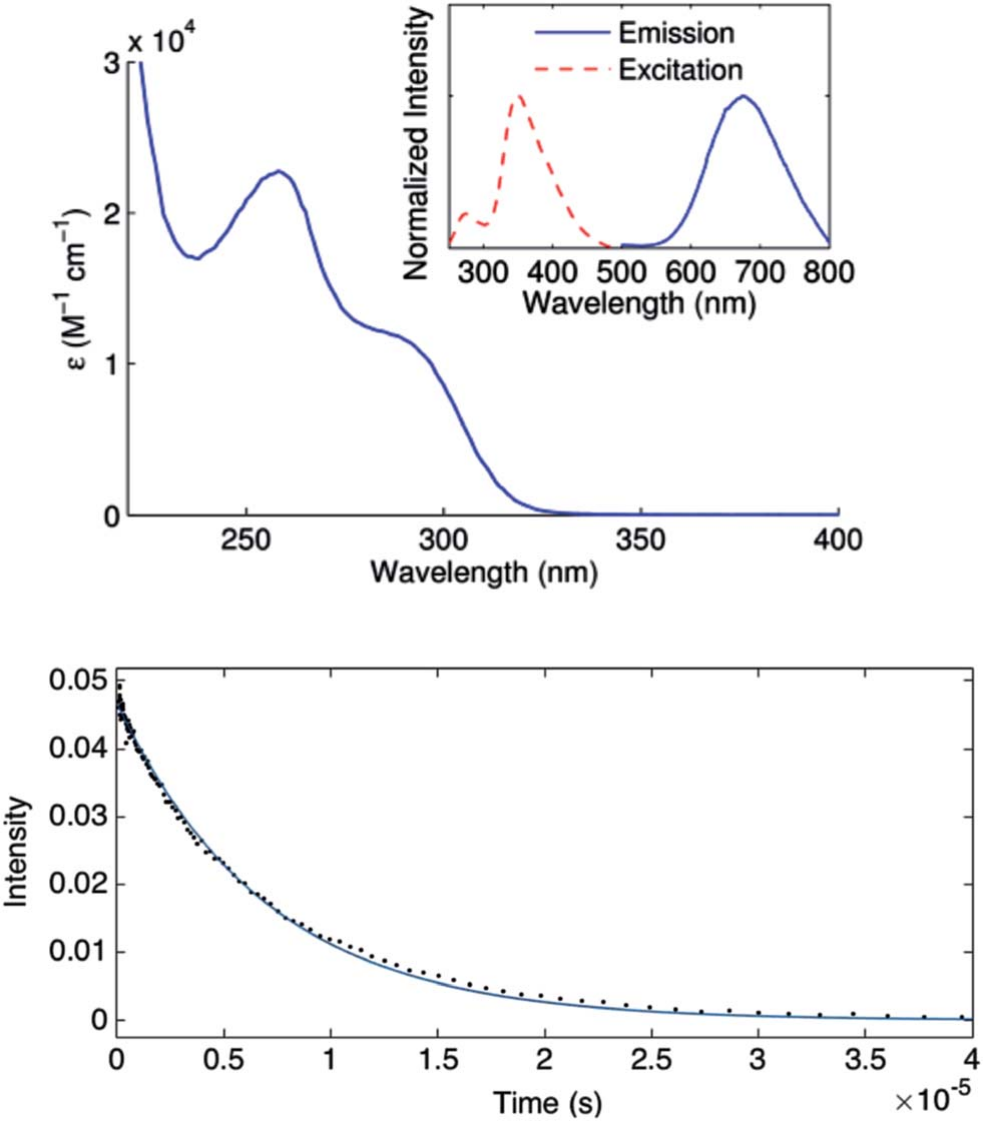
Top: Optical spectrum of [Cu^I^(SAr)_2_]Na (1) (*λ*_max_ = 258 nm, *ε* = 2.3 × 10^4^ cm^−1^ M^−1^); inset: excitation spectrum at 675 nm emission (dashed line) and emission spectrum at 353 nm excitation (solid line); in acetonitrile at 25 °C. Bottom: Time-resolved decay of the luminescence intensity of 1*; in acetonitrile at 25 °C (25 μM); Nd:YAG laser at 355 nm excitation; observation wavelength: 675 nm.

Complex 1 absorbs strongly in the ultraviolet region (top of Fig. 7), although only weakly at 365 nm (*ε*_365_ = 3 M^−1^ cm^−1^), a prominent emission band for the 100 watt Hg lamp used in our photoinduced C–S couplings.^26^ The complex luminesces upon excitation at 355 nm with a lifetime of ∼7 μs in acetonitrile, as determined by transient luminescence spectroscopy (bottom of Fig. 7). The lifetime of the emissive state does not change as a function of the observation wavelength, consistent with a single species being the source of luminescence. While the lack of a reversible Cu^I^/Cu^II^ redox couple precludes a true evaluation of the excited-state reduction potential for complex 1, we estimate this potential to be −2.5 to −2.7 V on the basis of the first ground-state oxidative feature (*E*_p_ = −0.18 V *vs.* SCE) (see Electrochemistry) and an approximate *E*^00^ of 2.3–2.5 eV.^27,28^ These data suggest that the excited state of complex 1 is sufficiently long-lived and reducing to engage in electron transfer with electrophiles such as aryl iodides (Ph–I: −1.91 V *vs.* SCE in DMF^21^).

**Fig. 8:**
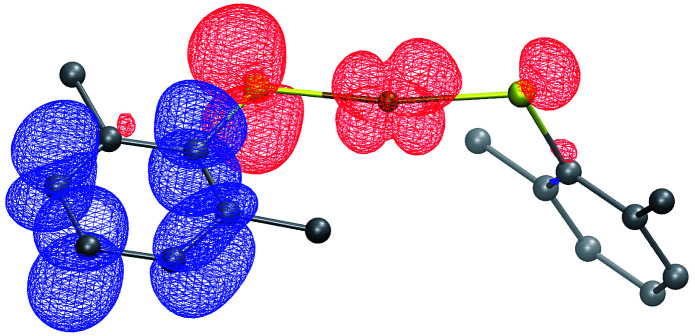
Difference density plot for the lowest energy absorption band of [Cu^I^(SAr)_2_]Na (1). The donor orbital is shown in red, and the acceptor orbital is shown in blue (isovalue = 0.02).

To gain insight into the predicted electronic structure of the excited state of complex 1, we have performed time-dependent DFT calculations.^29^ These calculations indicate that the lowest energy singlet state (*λ*_calc_ = 325 nm) consists of a transition from the HOMO (Cu–S antibonding) to the arene π* (Fig. 8). The population of a high-energy arene π* orbital in the excited state is consistent with 1 being a potent photoreductant.

### Stern–Volmer kinetic analysis

**Fig. 9:**
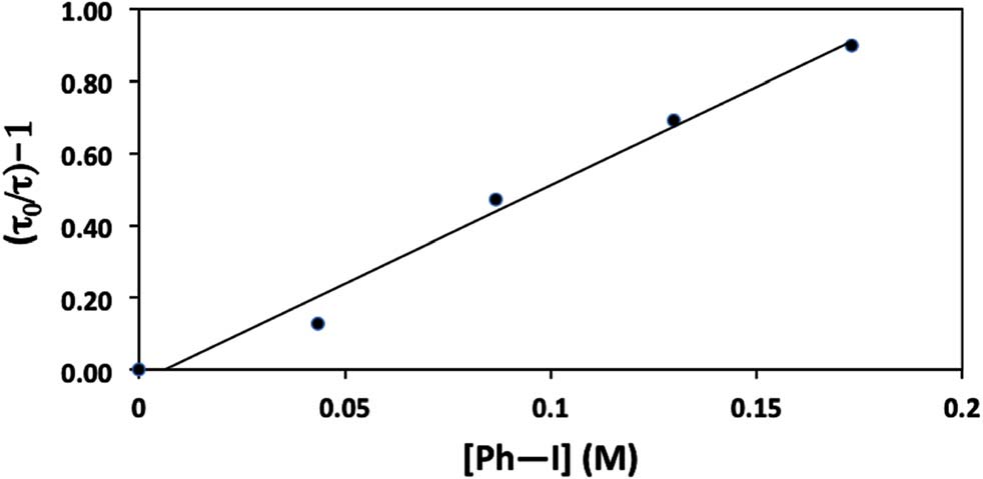
Stern–Volmer plot for the luminescence quenching of [Cu^I^(SAr)_2_]Na* in the presence of Ph–I.

The mechanisms outlined in Fig. 1–3 begin with electron transfer from a photoexcited copper(i)–thiolate (B) to the aryl halide. We have conducted a Stern–Volmer kinetic analysis of this elementary step, specifically, the reaction of the excited state of [Cu^I^(SAr)_2_]Na (1) with Ph–I (reduction potentials: [Cu^I^(SAr)_2_]^−^*: ∼−2.6 V; Ph–I: −1.91 V *vs.* SCE in DMF^21^), and we have determined that the rate constant for quenching is 8 × 10^5^ M^−1^ s^−1^. As expected, an increase in the concentration of Ph–I leads to a decrease in the lifetime of the excited state (Fig. 9). The observed quenching results from electron transfer, not energy transfer; the emission spectrum of complex 1 exhibits no overlap with the absorption spectrum of Ph–I.^30,31^

### Consideration of a radical chain mechanism: quantum yield and chain length

We have established that the quantum yield (*Φ*) for the stoichiometric coupling of [Cu^I^(SAr)_2_]Na (1) with Ph–I when irradiated at 365 nm is 0.08(2),^32^ a value that can be accommodated either by a non-chain mechanism or by a chain mechanism with rapid chain termination. By dividing the quantum yield by the Stern–Volmer quenching fraction (*Q*), we have determined the chain length (the number of molecules of product formed per photoinduced electron-transfer event) for the C–S coupling of complex 1 with Ph–I to be 0.8 (eqn (8)). This suggests that this cross-coupling proceeds *via* a non-chain pathway, as a chain mechanism would be expected to furnish more than one molecule of product from each photoinduced electron transfer. In contrast, Yoon concluded on the basis of a similar analysis that three representative reactions that involve photoredox catalysis proceed through a chain pathway.^9^8
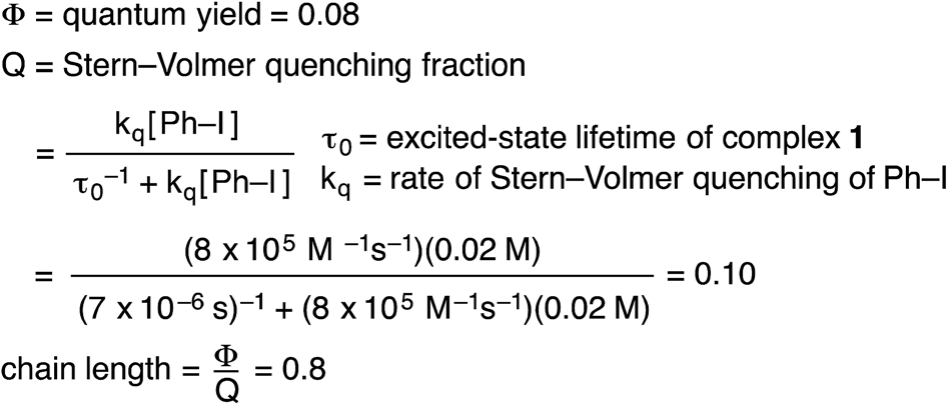


### Viability of coupling an aryl radical with a copper–thiolate

The mechanistic observations described above are consistent with the suggestion that an aryl radical is generated under our cross-coupling conditions. This intermediate could subsequently form a C–S bond by reacting with species such as a Cu(ii)–thiolate (Fig. 1) or a Cu(i)–thiolate (Fig. 2). We sought insight into the viability of such couplings by exploring reactions of an aryldiazonium salt, which can readily be converted into an aryl radical *via* one-electron reduction.^2*d*^

When [Cu^I^(SAr)_2_]Na (1; *E*_p_ = −0.18 V *vs.* SCE) and 4-methoxyphenyldiazonium tetrafluoroborate (*E*° = 0.14 V *vs.* SCE^33^) are allowed to react in CD_3_CN at −20 °C for 30 minutes, no coupling is evident by ^1^H NMR spectroscopy (Table 1, entry 1). However, upon warming the mixture to room temperature for 30 minutes, C–S bond formation proceeds in 57% yield (entry 2). One possible pathway for this transformation begins with electron transfer from complex 1 to the aryldiazonium salt to afford a copper(ii)–thiolate and Ar^1^–N_2_, which loses N_2_ to generate an aryl radical that couples with the copper(ii)–thiolate to form the C–S bond (eqn (9)).^34^

**Table 1:** Reactions of a copper–thiolate with an aryldiazonium salt

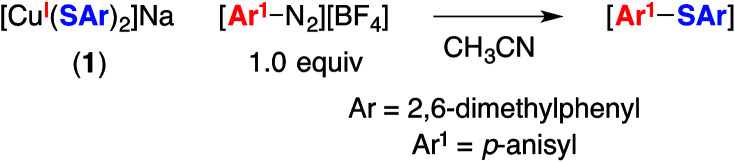		
Entry	Conditions	Yield (%)
1	−20 °C, 30 min	<2^a^
2	r.t., 30 min	57
3	1.1 equiv. FeCp^*^_2_, −20 °C, 30 min	22
4	r.t., 30 min, 1.1 equiv. [FeCp^*^_2_][BF_4_]	56

a Run in CD_3_CN; unreacted diazonium salt: >90%.


9

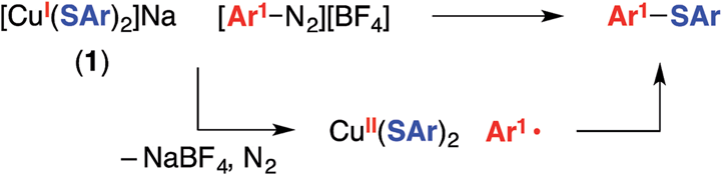



To assess the viability of the coupling of an aryl radical with a copper(i)–thiolate, we sought a reductant that would reduce the aryldiazonium salt and thereby generate an aryl radical under conditions in which copper(i)–thiolate 1 would not (CD_3_CN, −20 °C; Table 1, entry 1). We determined that, in the presence of decamethylferrocene (FeCp^*^_2_; *E*° = −0.12 V *vs.* SCE^35^),^36^ the aryldiazonium salt is completely consumed within 30 minutes at −20 °C, furnishing a mixture of compounds that includes a 22% yield of the C–S coupling product (entry 3). The low yield of the diarylsulfide indicates that under these conditions an aryl radical reacts inefficiently, at best, with a copper(i)–thiolate to form a C–S bond; control experiments suggest the alternative possibility that at least some of the cross-coupling product may be formed from reaction of the aryl radical with a small amount of copper(ii)–thiolate that is generated through a redox equilibrium between Cu^I^/Fe^III^ and Cu^II^/Fe^II^ as the ferrocenium ion is formed.^37^ When the coupling illustrated in entry 2 is conducted in the presence of [FeCp^*^_2_][BF_4_] (entry 4), the yield of the diarylsulfide is essentially unchanged (56%; entry 2 *versus* entry 4). This result indicates that the ferrocenium ion that is produced in entry 3 is not responsible for the diminished yield in that reaction.

### Rate of capture of an aryl radical by a copper–thiolate; in-cage *versus* out-of-cage coupling

To obtain insight into the rate of capture of the aryl radical intermediate, we have determined the ratio of uncyclized/cyclized products for C–S couplings of several aryl iodides that have previously been employed in radical-clock studies (Table 2).^38,39^ Our data indicate that capture of the aryl radical by a copper–thiolate occurs competitively with a cyclization process that has a first-order rate constant of ∼4 × 10^8^ s^−1^ in benzene.

**Table 2:** Reaction of an aryl radical: cyclization *versus* capture by a copper–thiolate

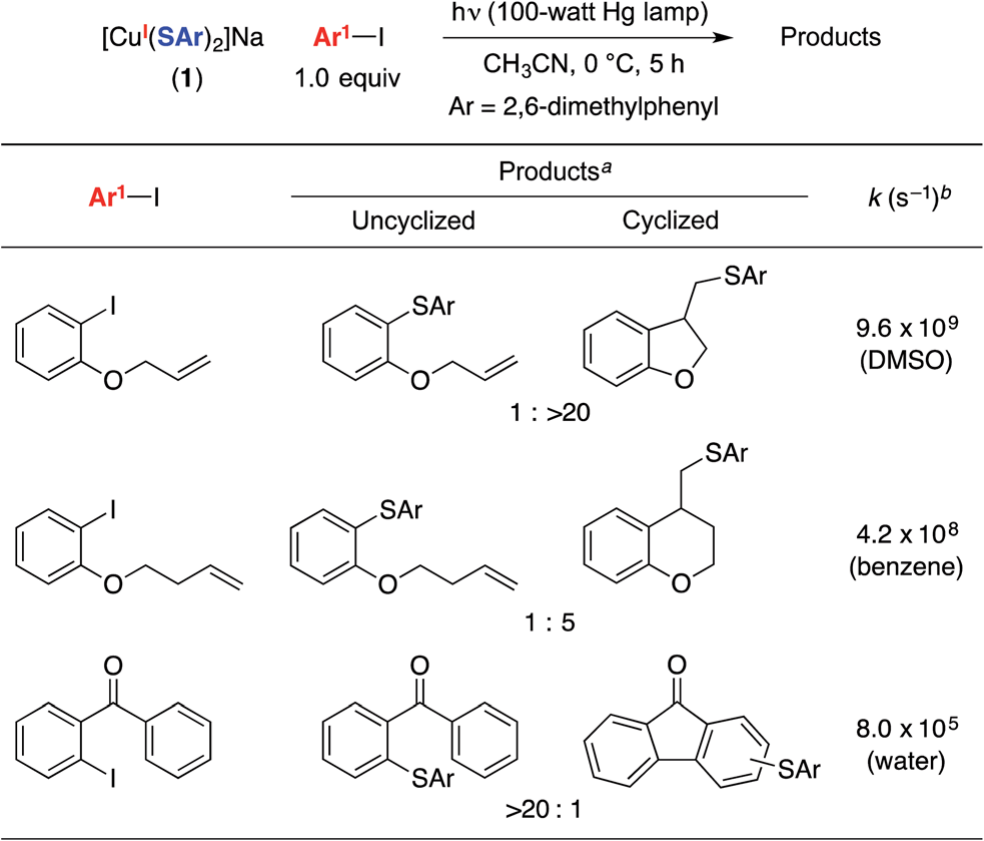

a Ratio determined through GC analysis with dodecane as an internal standard (average of two experiments).

b Rate of cyclization of the aryl radical (20–25 °C).

We have examined the relationship between the amount of [Cu^I^(SAr)_2_]Na (1) and the ratio of uncyclized/cyclized products, and we have determined that the product ratio remains essentially constant as we alter the quantity of complex 1 or the overall concentration (eqn (10)). These observations can be accommodated by the mechanism illustrated in Fig. 1, if C–S bond formation occurs between the aryl radical and copper(ii)–thiolate C within the solvent cage (*i.e.*, a single copper complex serves first as the electron donor and then as the source of SAr). In contrast, for the mechanism illustrated in Fig. 2, the cyclized/uncyclized product ratio should depend on parameters such as stoichiometry and concentration, since C–S bond formation requires the aryl radical to leave the solvent cage and to encounter a copper(i)–thiolate (*i.e.*, one copper complex serves as the electron donor and a different copper complex provides the SAr group).^40,41^10
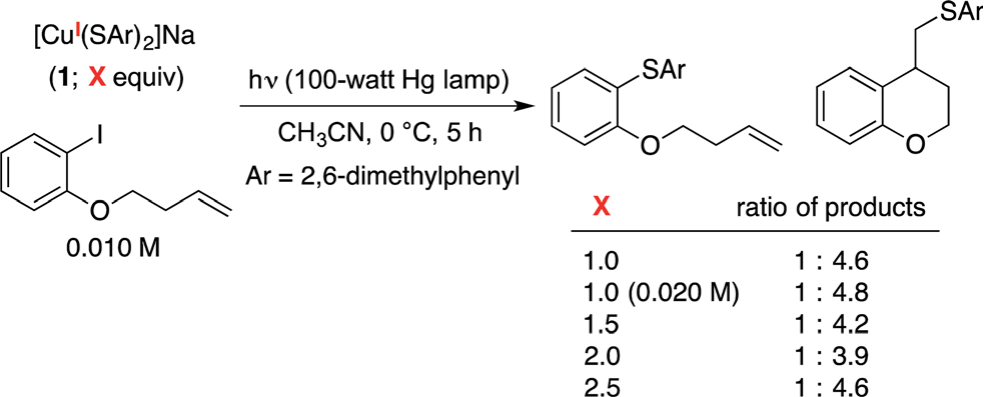


### Spectroscopic evidence for a copper(ii)–thiolate

**Fig. 10:**
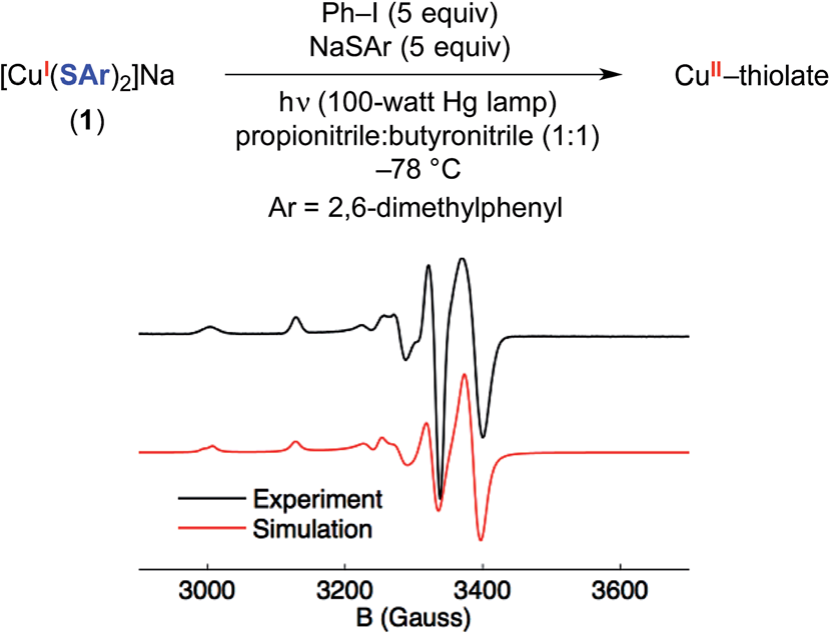
X-band EPR spectrum (77 K) of a coupling reaction following irradiation for 5 min. Simulation parameters: *g*_1_ = 2.022, *g*_2_ = 2.032, *g*_3_ = 2.104, *A*_1_(Cu) = 85 MHz, *A*_2_(Cu) = 130 MHz, and *A*_3_(Cu) = 360 MHz.

As noted above, electron transfer from an excited-state copper(i)–thiolate (B) to an aryl halide to generate a copper(ii)–thiolate (C) is a key step in several of the mechanisms under consideration. Copper(ii) species are *S* = 1/2 and therefore readily detected by EPR spectroscopy, as is the case for copper(ii)–thiolate complexes.^42^ Indeed, photolysis of a solution of [Cu^I^(SAr)_2_]Na (1) in the presence of excess Ph–I and NaSAr in propionitrile : butyronitrile (1 : 1) at −78 °C results in a blue solution, the EPR spectrum of which is consistent with the presence of some amount of a copper(ii)–thiolate radical (Fig. 10).^43^

The four-line hyperfine coupling is consistent with an *I* = 3/2 paramagnetic copper complex with a single metal center. The spectrum shows modest *g* anisotropy compared to other copper(ii)–thiolate complexes,^42^ which is consistent with a highly covalent Cu–S interaction.^44^ This suggests that significant radical character resides in sulfur p orbitals, and DFT calculations support this assessment (see below).

**Fig. 11:**
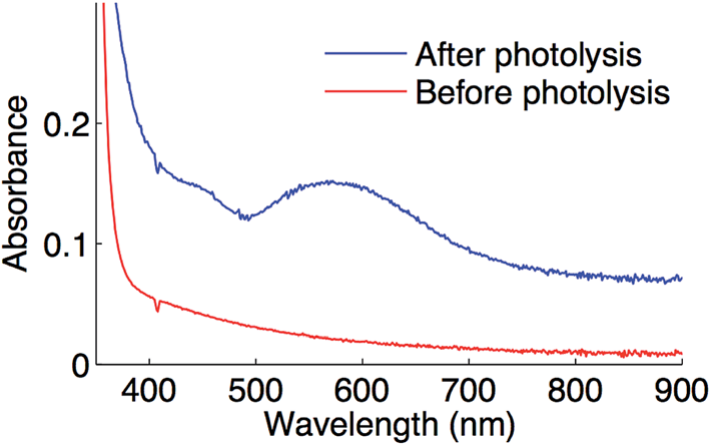
Optical spectrum of a coupling reaction prior to photolysis (red) and after photolysis (blue) in propionitrile at −78 °C.

Optical spectroscopy can serve as an additional technique for characterizing the putative copper(ii)–thiolate. Upon irradiating complex 1 in the presence of Ph–I and NaSAr in propionitrile at −78 °C, a feature at 582 nm is observed (Fig. 11), which is consistent with the blue color of the reaction mixture. This feature is near the range found for sulfur-rich copper(ii) proteins (593 to 610 nm).^45^

While the above data provide strong evidence for the generation of a copper(ii)–thiolate radical upon photolysis of a mixture of complex 1, Ph–I, and NaSAr, they do not identify the specific paramagnetic copper species, and further characterization is complicated by its instability even at −78 °C. The presence not only of Ph–I, but also of NaSAr, is required for detection of this copper(ii)–thiolate radical by optical and by EPR spectroscopy. In view of the need for exogenous thiolate, we hypothesize that a copper(ii) tris(thiolate), [Cu^II^(SAr)_3_]^−^, may be formed, *e.g.*, *via* electron transfer from [Cu^I^(SAr)_2_]^−^* to the aryl halide to form Cu^II^(SAr)_2_, followed by trapping by NaSAr (eqn (11)).^46^ DFT calculations suggest that binding of an arylthiolate to Cu^II^(SAr)_2_ is exergonic by ∼4 kcal mol^−1^.^47,48^11
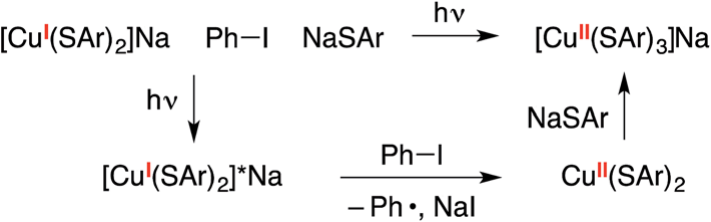


Alternatively, the copper(ii) tris(thiolate), [Cu^II^(SAr)_3_]^−^, could be generated by electron transfer to Ph–I from the excited state of NaSAr, followed by coupling of the thiyl radical with [Cu^I^(SAr)_2_]^−^ (eqn (12)). Our observations to date do not allow us to definitively distinguish between these two pathways for the formation of putative [Cu^II^(SAr)_3_]^−^.12
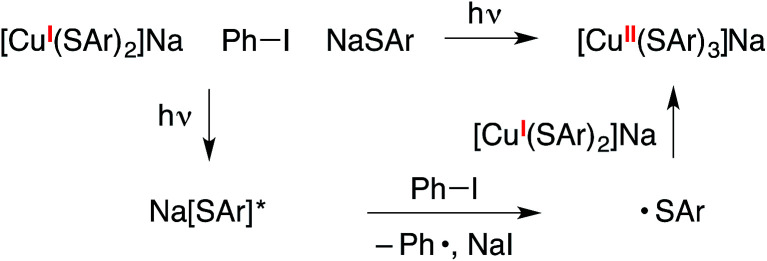


**Fig. 12:**
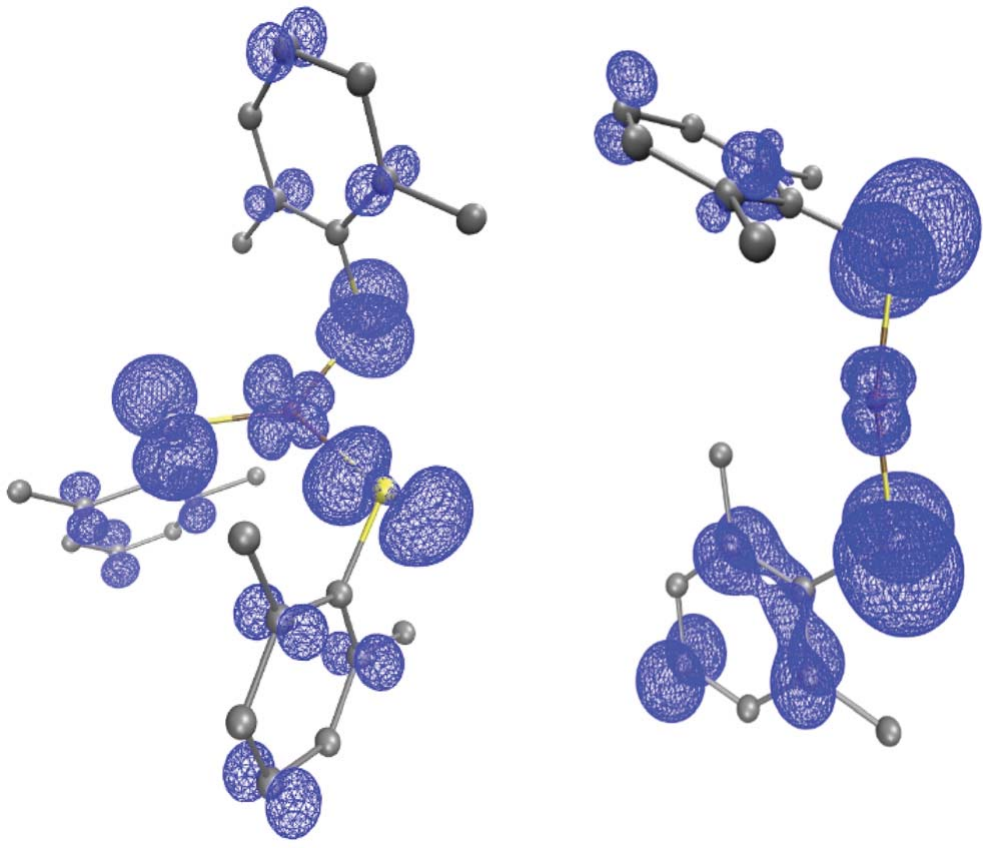
Spin density plots (0.002 isocontours) of [Cu^II^(SAr)_3_]^−^ (left) and Cu^II^(SAr)_2_ (right).

DFT calculations of [Cu^II^(SAr)_3_]^−^ and of Cu^II^(SAr)_2_ predict that significant spin density would reside on the thiolate ligands for either compound, which suggests that C–S bond formation could occur through direct reaction of the aryl radical with the copper-bound thiolate ([Cu^II^(SAr)_3_]^−^: Cu 0.23e^−^, 3S 0.57e^−^; Cu^II^(SAr)_2_: Cu 0.14e^−^, 2S 0.63e^−^; Fig. 12).

## Conclusions

In this report, we describe the first detailed mechanistic investigation of one of the photoinduced, copper-mediated cross-couplings that we have developed, specifically, the coupling of a thiol with an aryl iodide. Due to the existence of a parallel, copper-free C–S bond-forming pathway, we have focused our attention on understanding the stoichiometric chemistry of a key proposed intermediate, [Cu^I^(SAr)_2_]^−^ (Ar = 2,6-dimethylphenyl); our observations to date are consistent with the viability of the elementary steps outlined in Fig. 1 (A → D).

We have established that [Cu^I^(SAr)_2_]Na (1) is a two-coordinate monomer both in the solid state and in solution, and we have detected [Cu^I^(SAr)_2_]^−^ through ESI-MS under cross-coupling conditions. Complex 1 undergoes excitation upon irradiation at 365 nm (a prominent emission band of a Hg lamp), and it luminesces with a lifetime of ∼7 μs; we estimate its excited-state reduction potential to be ∼–2.6 V. Through a Stern–Volmer study, we have determined that the excited state is effectively quenched by Ph–I, as expected on the basis of its reduction potential; correspondingly, complex 1 reacts with Ph–I upon irradiation to afford the C–S coupling product. By employing actinometry, we have established that the chain length for the coupling of complex 1 with Ph–I is 0.8, indicating a non-chain mechanism. Our EPR and optical spectroscopy data suggest that a copper(ii)–thiolate is formed when complex 1 is irradiated in the presence of Ph–I and NaSAr. Furthermore, through the use of an aryldiazonium salt, we have independently generated an aryl radical in the presence of copper(i)– and copper(ii)–thiolates, and we have provided evidence that C–S bond formation is more efficient in the case of a copper(ii)–thiolate. Finally, with the aid of radical clocks, we have established that C–S bond formation likely occurs *via* an in-cage mechanism in which a single copper complex serves both as an electron donor (Cu^I^ → Cu^II^) and a source of SAr (copper(ii)–thiolate). Thus, the available data support the viability of the elementary steps for photoinduced C–S coupling that are illustrated in Fig. 1 (A → D), a mechanism that is distinct from most applications of photoredox catalysts in organic synthesis; other C–S coupling pathways, for example involving initial photoreduction of the aryl halide by a photoexcited copper-free thiolate, may also be operative. Our current efforts are directed at evaluating the degree to which the mechanism illustrated in Fig. 1, or alternative mechanisms, applies to other photoinduced, copper-catalyzed cross-couplings.

## Supplementary Material

SC-007-C5SC04709A-s001

SC-007-C5SC04709A-s002

SC-007-C5SC04709A-s003
