# Supplementary material for: A mechanistic investigation of the photoinduced, copper-mediated cross-coupling of an aryl thiol with an aryl halide
Source: Chem Sci. 2016 Feb 24;7(7):4091–100. doi: 10.1039/c5sc04709a (PMC5201211; doi:10.1039/c5sc04709a)

Electronic Supplementary Material (ESI) for Chemical Science.
This journal is © The Royal Society of Chemistry 2016

# A Mechanistic Investigation of the Photoinduced, Copper-Mediated Cross-Coupling of an Aryl Thiol with an Aryl Halide

**Miles W. Johnson, Kareem I. Hannoun, Yichen Tan, Gregory C. Fu,* and Jonas C. Peters***

*Division of Chemistry and Chemical Engineering, California Institute of Technology, Pasadena, California 91125, United States*

**Supporting Information**

**Table of Contents**

I. General Considerations ---------- S-2
II. Synthesis and Characterization ---------- S-5
III. Molar Conductivity Measurements ---------- S-9
IV. Spectroscopic Observation of Copper(II) Thiolate ---------- S-10
V. Identification of $[Cu^I(SAr)_2]^-$ by ESI-MS ---------- S-11
VI. Radical Clock Experiments ---------- S-12
VII. Reactivity of $[Cu^I(SAr)_2]Na$ ---------- S-14
VIII. Stern-Volmer Quenching Experiment ---------- S-15
IX. Steady-State Fluorimetry Experiment ---------- S-16
X. Reactivity of 1-(but-3-en-1-yloxy)-2-iodobenzene with $[Cu^I(SAr)_2]Na$ ---------- S-17
XI. VT-NMR Study of $[Cu^I(SAr)_2]Na$ ---------- S-18
XII. DOSY Analysis ---------- S-19
XIII. Actinometric Study ---------- S-20
XIV. Emission Spectrum of 100-W Hg Lamp ---------- S-22
XV. Determination of Molar Absorptivities (ε) ---------- S-23
XVI. Absorption Spectra of $[Cu^I(SAr)_2]Na$ in the Presence of Sodium 2,6-dimethylthiophenolate ---------- S-24
XVII. DFT Calculations ---------- S-25
XVIII. Probe of Direct Coupling between $[Cu^I(SAr)_2]Na$ and Aryl Radical ---------- S-27
XIX. Cage Escape Efficiency as a Function of Temperature ---------- S-29
XX. Calculation of Excited State Reduction Potential of 1 ---------- S-30
XXI. References ---------- S-31
XXII. X-Ray Crystallographic Data ---------- S-32
XXIII. $^1H$ and $^{13}C$ NMR Data ---------- S-33

## I. General Considerations

**Chemicals.** Toluene, acetonitrile, and diethyl ether were degassed with nitrogen and dried by passage through activated alumina using a solvent purification system. Acetonitrile used in photophysical studies was purchased from Alfa Aesar (HPLC Grade, 99.9%+), degassed by three freeze-pump-thaw cycles, and passed through activated alumina prior to use. Phenyl halides were stored over 4 Å molecular sieves and passed through activated alumina prior to use. The following compounds were synthesized according to literature procedures: mesitylcopper,[1] 3-methyl-2,3-dihydrobenzofuran,[2] 1,2-bis(2,6-dimethylphenyl) disulfide,[3] 1-(allyloxy)-2-iodobenzene,[4] 2,6-dimethylphenyl phenyl sulfide,[5] 2-(allyloxy)benzenediazonium tetrafluoroborate,[6] [$n$-$Bu_4N$][$B(C_6F_5)_4$],[7] 1-hydroxyl-2,2,6,6-tetramethylpiperidine,[8] and 1-(but-3-en-1-yloxy)-2-iodobenzene.[9] All other chemicals were purchased from commercial suppliers.

**Infrared, EPR, and UV-Vis Spectroscopy.** UV-Vis experiments were conducted with sealable 1-cm path length fused quartz cuvettes (Starna Cells) using a Cary 50 UV-Vis spectrometer equipped with a UNISOKU Scientific Instruments Coolspek cryostat. X-band EPR measurements were made with a Bruker EMX spectrometer at 77 K. Simulation of EPR data was conducted using the software EasySpin.[10] IR measurements were recorded on a Bruker ALPHA Diamond ATR.

**NMR Spectroscopy.** All NMR spectra were obtained at ambient temperature using Varian 400 and 500 MHz spectrometers unless otherwise noted. $^1H$ NMR chemical shifts ($\delta$) are reported in parts per million (ppm) relative to the proteo solvent impurity (7.26 ppm for $CHCl_3$, 1.94 ppm for $CD_2HCN$). $^{13}C$ NMR chemical shifts were also reported relative to the solvent peak (77.16 for $CDCl_3$).

**Mass Spectrometry.** The ESI-MS for **1** was conducted using a Thermo LCQ ion trap mass spectrometer. Mass spectral data for all organic compounds were collected on an Agilent 5973.

**Photophysical Methods.** Time-resolved luminescence measurements were conducted using a Q-switched Nd:YAG laser (Spectra-Physics Quanta-Ray PRO-Series) with 8 ns pulses (repetition rate of 10 Hz) in the Beckman Institute Laser Resource Center at the California Institute of Technology (http://www.bilrc.caltech.edu). The luminescence was dispersed through a monochromator onto a photomultiplier tube (Hamamatsu R928). Samples were stirred continuously. Steady-state emission spectra were recorded on a Jobin Yvon Spec Fluorolog-3-11. Sample excitation was accomplished with a xenon arc lamp and the right angle emission detected with a photomultiplier tube (Hamamatsu R928P). All measurements were conducted with 1-cm path length fused quartz cuvettes (Starna Cells).

**Cyclic Voltammetry**. Electrochemical experiments were performed in acetonitrile with 0.1 M [$n$-$Bu_4N$][$B(C_6F_5)_4$] as the electrolyte in a nitrogen-filled glovebox. A CH

600B potentiostat was used with a glassy carbon working electrode and a platinum wire auxiliary electrode. The reference electrode was a Ag/$AgNO_3$ (0.1 mM)/acetonitrile reference electrode containing 0.1 M [$n$-$Bu_4N$][$B(C_6F_5)_4$] and separated from the solution by a Vycor frit. The reference electrode was externally referenced to ferrocene. All reported potentials were determined against the reference electrode and converted to SCE by adjusting by 380 mV.

**Photolytic Reactions.** Photolytic reactions were performed using a 100-W Blak-Ray Long Wave Ultraviolet Lamp (Hg), 100-W Blak-Ray B-100Y High Intensity Inspection Lamp (Hg), or a Luzchem LZC-4V photoreactor equipped with LZC-UVA lamps centered around 350 nm. Temperature control was maintained with either an ice water bath, or an isopropanol bath cooled by an SP Scientific cryostat. For reactions using mercury lamps, the light source was placed approximately 20 cm above the sample and the reaction mixture was stirred vigorously using a magnetic stir bar. All reactions were performed in VWR 16 x 100 mm borosilicate culture tubes that were capped with septa and electrical tape. Punctures in the septa were sealed with vacuum grease.

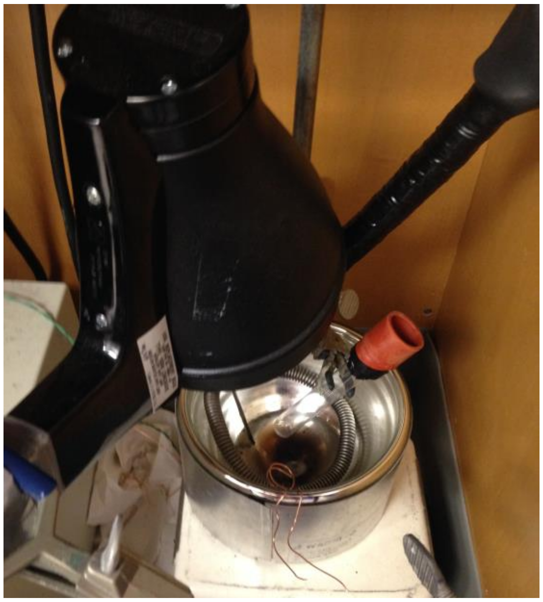

**Figure S1.** Representative reaction set-up using a 100-W Hg lamp. Ice is excluded for clarity.

**Chromatography.** Normal phase column chromatography was performed using Silicycle 230-400 mesh silica gel. Analytical thin layer chromatography was conducted with Merck aluminum-backed TLC plates (silica gel 60 F254) and plates were visualized under UV light. Reverse-phase chromatography was performed with a Biotage Isolera Spektra Four system.

**Other Characterization Methods.** Elemental analysis was performed by Midwest Microlab, LLC. Calibrated GC yields were obtained using an Agilent 6890N gas chromatograph (FID detector) with dodecane as an internal standard.

**X-ray Crystallography.** XRD studies were carried out at the Beckman Institute Crystallography Facility (http://www.its.caltech.edu/~xray/index.html) on a Bruker D8 Venture kappa duo photon 100 CMOS instrument (Mo Kα radiation). Structures were solved using SHELXT and refined against $F^2$ by full-matrix least squares with SHELXL and OLEX2. Hydrogen atoms were added at calculated positions and refined using a riding model. The crystals were mounted on a glass fiber or a nylon loop with Paratone N oil.

## II. Synthesis and Characterization

*Reported yields have not been optimized.*

**General Procedure A:** This procedure is a modification of that developed by Peters, Fu, and co-workers.[5] In a nitrogen-filled glovebox, electrophile, NaO*t*-Bu, CuI, and acetonitrile were added to a borosilicate tube. The tube was then capped with a septum and sealed with electrical tape. On a Schlenk line, 2,6-dimethylthiophenol was added *via* syringe. The vessel was then immersed in a cooling bath and irradiated for the specified period. The reaction mixture was then concentrated and the crude material extracted in diethyl ether and filtered through a thin pad of silica. Following concentration, the material was purified by column chromatography.

**General Procedure B:** The method developed by Venkataraman and coworkers was used for the independent synthesis of various diarylthioethers as indicated below.[11] In a nitrogen-filled glovebox, a borosilicate test tube or round bottom flask was charged with CuI (10 mol%), neocuproine (DMPHEN) or its hemihydrate (10 mol%), aryl iodide (1 equiv.), NaO*t*-Bu (1.5 equiv.), and toluene. The reaction vessel was removed from the glovebox and connected to a Schlenk line. The reaction mixture was charged with 2,6-dimethylthiophenol (1.1 equiv.) *via* syringe. The reaction mixture was heated at 105 to 110 °C for the specified time, cooled to room temperature, and filtered. The crude material was purified by column chromatography.

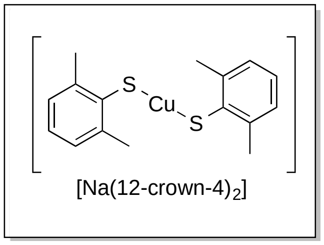


**Copper(I) bis(2,6-dimethylthiophenolate) sodium bis(12-crown-4) ($[Cu^I(SAr)_2]Na$)** A Schlenk bomb was charged with NaO*t*-Bu (90.7 mg, 0.943 mmol), mesitylcopper (181 mg, 0.991 mmol), and acetonitrile (4 mL) in the glovebox. The bomb was removed from the glovebox and connected to a Schlenk line. 2,6-dimethylthiophenol (250 μL, 1.88 mmol) was added *via* syringe, causing the orange suspension to turn white. The reaction mixture was stirred for 30 min, after which time the bomb was returned to the glovebox and its contents filtered through a plug of Celite. 12-crown-4 (162 μL, 1.00 mmol) was added to the filtrate, inducing precipitation of a white solid. The supernatant was removed *via* pipette and the solid was washed with diethyl ether. The desired product was isolated as an analytically pure white solid (214 mg, 0.300 mmol, 32% yield) following removal of solvent *in vacuo*. X-ray quality crystals were grown from an acetonitrile solution at ambient temperature over 12 h. $^1$H NMR (400 MHz, $CD_3CN$): δ 6.91 (d, *J* = 7.4 Hz, 4H), 6.70 (t, *J* = 7.4 Hz, 2H), 3.62 (s, 32H), 2.42 (s, 12H). HR-MS (ESI) (*m*/*z*) calcd for $[C_{16}H_{18}CuS_2]^-$: 337.0146, found: 337.1133. Calculated for $C_{32}H_{50}CuNaO_8S_2$: C, 53.88; H, 7.06. Found: C, 53.69, H, 7.14. UV-Vis (MeCN): $\lambda_{max}$ = 258 nm, ε = 2.3 × $10^4$ $M^{-1}$ $cm^{-1}$.

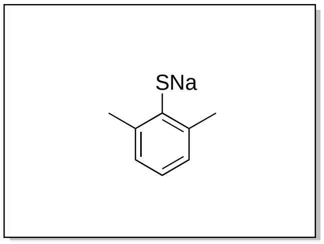

**Sodium 2,6-dimethylthiophenolate** A Schlenk flask was charged with oil-free sodium hydride (175 mg, 7.29 mmol) and diethyl ether (20 mL) in a glovebox and the vessel was sealed with a septum. The reaction mixture was cooled to 0 °C and 2,6-dimethylthiophenol (1.00 mL, 7.51 mmol) was delivered to the suspension *via* syringe on a Schlenk line. White precipitate immediately formed. Following stirring for 48 h at ambient temperature, the solvent was transferred via cannula from the flask and the solid was then triturated with pentane (*ca*. 100 mL). The desired product was isolated as a spectroscopically pure white solid (1.05 g mg, 6.52 mmol, 89% yield) following removal of solvent *in vacuo*. $^1$H NMR (400 MHz, $CD_3CN$) δ 6.80 (d, *J* = 7.3 Hz, 2H), 6.44 (t, *J* = 7.5 Hz, 1H), 2.30 (s, 6H). UV-vis (MeCN): $\lambda_{max}$ = 292 nm, ε = 1.9 × $10^4$ $M^{-1}$ $cm^{-1}$.

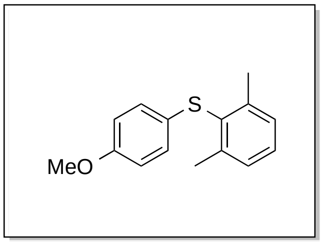

**4-Methoxyphenyl 2,6-dimethylphenyl sulfide** According to General Procedure B, CuI (42.0 mg, 0.22 mmol), DMPHEN hemihydrate (43.6 mg, 0.20 mmol), 4-iodoanisole (463 mg, 1.98 mmol), 2,6-dimethylthiophenol (280 μL, 2.21 mmol), toluene (6 mL) and NaO*t*-Bu (293 mg, 3.05 mmmol) were heated for 48 h. The product was isolated as a white solid (251 mg, 1.03 mmol, 52% yield) following column chromatography ($SiO_2$, 4% EtOAc:hexanes). $^1$H NMR (400 MHz, $CDCl_3$): δ 7.23 – 7.12 (m, 3H), 6.91 (d, *J* = 8.7 Hz, 2H), 6.75 (d, *J* = 8.7 Hz, 2H), 3.75 (s, 3H), 2.43 (s, 6H). $^{13}$C NMR (101 MHz, $CDCl_3$): δ 157.6, 143.6, 132.0, 129.0, 128.7, 128.5, 128.0, 114.8, 55.4, 22.1. LR-MS (EI) (*m*/*z*) calculated for $[C_{15}H_{19}OS]^+$: 244.1, found: 244.1. FT-IR (thin film): 3059, 2954, 2832, 1592, 1572, 1490, 1459, 1439, 1283, 1238, 1173, 1032, 820, 769, 638, 625, 536, 507 $cm^{-1}$.

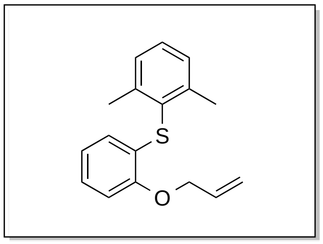

**2-(Allyloxy) 2,6-dimethylphenyl sulfide** According to General Procedure B, CuI (19.3 mg, 0.10 mmol), DMPHEN hemihydrate (22.1 mg, 0.10 mmol), 1-(allyloxy)-2-iodobenzene (251 mg, 0.96 mmol), 2,6-dimethylthiophenol (140 μL, 1.10 mmol), toluene (6 mL) and NaO*t*-Bu (147 mg, 1.5 mmmol) were heated for 14 h. The product was isolated as a white solid (178 mg, 0.658 mmol, 69% yield) by column chromatography ($SiO_2$, 4% EtOAc:hexanes). $^1$H NMR (400 MHz, $CDCl_3$) δ 7.30 – 7.14 (m, 3H), 7.07 – 6.97 (m, 1H), 6.84 (d, *J* = 8.1 Hz, 1H), 6.72 (td, *J* = 7.6, 1.2 Hz, 1H), 6.33 (dd, *J* = 7.8, 1.6 Hz, 1H), 6.11 (ddt, *J* = 17.4, 10.3, 5.0 Hz, 1H), 5.51 (dq, *J* = 17.3, 1.7 Hz, 1H), 5.32 (dq, *J* = 10.6, 1.6 Hz, 1H), 4.67 (dt, *J* = 5.1, 1.7 Hz, 2H), 2.42 (s, 6H). $^{13}$C NMR (101 MHz, $CDCl_3$): δ 154.6, 144.5, 133.3, 129.8, 129.3, 128.6, 127.1, 125.2, 124.9, 121.6, 117.6, 112.1, 69.6, 21.9. LR-MS (EI) (*m*/*z*)

calculated for $[C_{17}H_{18}OS]^{+}$: 270.1, found: 270.1. FT-IR (thin film): 3059, 3017, 2955, 2920, 2894, 1575, 1474, 1439, 1233, 1103, 1040, 994, 919, 767, 742 $cm^{-1}$.

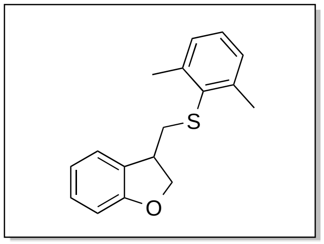

**3-(2,6-dimethylphenylthiomethyl)-2,3-dihydrobenzofuran** According to General Procedure A, CuI (15.7 mg, 0.082 mmol), NaO*t*-Bu (76.5 mg, 0.796 mmol), 1-(allyloxy)-2-iodobenzene (208 mg, 0.800 mmol), 2,6-dimethylthiophenol (102 μL, 0.800 mmol), and acetonitrile (2.5 mL) were combined and photolyzed with a mercury lamp for 19 h at -20 °C. The reaction mixture was concentrated, and the resulting material suspended in diethyl ether and filtered to remove insoluble byproducts. The product was isolated as a pale yellow oil (96.6 mg, 0.358 mmol, 45% yield) by column chromatography on silica gel (0 → 2% EtOAc/hexanes), followed by column chromatography using reverse-phase C-18 silica gel (0 → 100% acetonitrile/water). Due to co-elution of the title compound and its uncyclized isomer despite multiple attempts at purification, < 2% of the contaminant is detectable by GC and $^{1}$H NMR analysis. $^{1}$H NMR (400 MHz, $CDCl_3$): δ 7.19 (d, *J* = 7.4 Hz, 1H), 7.13 (m, 4H), 6.85 (td, *J* = 7.4, 1.0 Hz, 1H), 6.79 (d, *J* = 8.0 Hz, 1H), 4.63 (t, *J* = 9.0 Hz, 1H), 4.45 (dd, *J* = 9.1, 5.8 Hz, 1H), 3.63 – 3.45 (m, 1H), 3.06 (dd, *J* = 12.6, 4.6 Hz, 1H), 2.75 (dd, *J* = 12.7, 10.0 Hz, 1H), 2.56 (s, 6H). $^{13}$C NMR (101 MHz, $CDCl_3$): δ 160.0, 143.0, 133.0, 129.4, 128.9, 128.5, 128.4, 124.5, 120.6, 109.9, 76.3, 42.6, 39.9, 22.2. . LR-MS (EI) (*m/z*) calculated for $[C_{17}H_{18}OS]^{+}$: 270.1, found: 270.1. FT-IR (thin film): 3056, 2952, 2923, 2877, 1582, 1488, 1459, 1221, 1023, 772, 753 $cm^{-1}$.

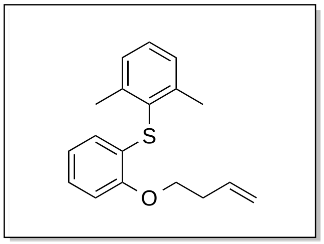

**2-(but-3-en-yloxy) 2,6-dimethylphenyl sulfide** According to General Procedure B, CuI (20.9 mg, 0.11 mmol), DMPHEN (11.0 mg, 0.0528 mmol), 1-(but-3-en-1-yloxy)-2-iodobenzene (136 mg, 0.990 mmol), 2,6-dimethylthiophenol (140 μL, 0.496 mmol), toluene (3 mL) and NaO*t*-Bu (73.2 mg, 0.761 mmmol) were heated for 16 h. The product was isolated as a colorless oil (101 mg, 0.354 mmol, 54% yield) following column chromatography ($SiO_2$, 1 → 2% EtOAc/hexanes). $^{1}$H NMR (400 MHz, $CDCl_3$) δ 7.34 – 7.18 (m, 3H), 7.07 (ddd, *J* = 8.0, 7.3, 1.6 Hz, 1H), 6.88 (dd, *J* = 8.1, 1.3 Hz, 1H), 6.75 (td, *J* = 7.5, 1.2 Hz, 1H), 6.40 (dd, *J* = 7.7, 1.6 Hz, 1H), 6.04 (ddt, *J* = 17.0, 10.2, 6.8 Hz, 1H), 5.35 – 5.12 (m, 2H), 4.17 (t, *J* = 6.8 Hz, 2H), 2.68 (qt, *J* = 6.8, 1.4 Hz, 2H), 2.47 (s, 6H). $^{13}$C NMR (101 MHz, $CDCl_3$) δ 154.9, 144.3, 134.5, 129.9, 129.2, 128.5, 127.0, 125.3, 124.9, 121.4, 117.2, 111.8, 68.3, 33.8, 21.8. LR-MS (EI) (*m/z*) calculated for $[C_{18}H_{20}OS]^{+}$: 284.1, found: 284.3. FT-IR (thin film): 3058, 2922, 1576, 1462, 1441, 1237, 1041, 1027, 918, 771, 743 $cm^{-1}$.

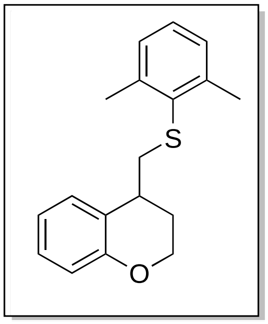

**4-(methylchromane) 2,6-dimethylphenyl sulfide** According to General Procedure A, CuI (9.0 mg, 0.047 mmol), 1-(allyloxy)-2-iodobenzene (122 mg, 0.445 mmol), 2,6-dimethylthiophenol (66.0 μL, 0.496 mmol), and NaO*t*-Bu (47.0 mg, 0.489 mmol) in acetonitrile (5 mL) were photolyzed with 350 nm light in a photobox at ambient temperature for 18 h. The product was isolated as a colorless semi-solid (24.0 mg, 0.084 mmol, 19% yield) by column chromatography on silica gel (0 → 2% EtOAc/hexanes), followed by column chromatography using reverse-phase C-18 silica gel (0 to 100% acetonitrile/water). $^1$H NMR (400 MHz, $CDCl_3$): δ 7.14 (ap s, 3H), 7.09 (tdd, *J* = 7.3, 1.9, 0.6 Hz, 2H), 7.02 – 6.94 (m, 1H), 6.88 – 6.73 (m, 1H), 4.30 – 4.20 (m, 1H), 4.20 – 4.09 (m, 1H), 3.12 – 3.04 (m, 1H), 2.96 – 2.87 (m, 1H), 2.88 – 2.79 (m, 1H), 2.59 (d, *J* = 0.7 Hz, 6H), 2.31 – 2.23 (m, 1H), 2.22 – 2.08 (m, 1H). $^{13}$C NMR (101 MHz, $CDCl_3$): δ 154.6, 142.8, 133.6, 129.2, 128.2, 127.8, 124.7, 120.3, 116.9, 62.9, 41.7, 33.9, 26.0, 22.1. LR-MS (EI) (*m*/*z*) calculated for $[C_{18}H_{20}OS]^+$: 284.1, found: 284.4. FT-IR (thin film): 3052, 2951, 2919, 1596, 1480, 1459, 1230, 965, 771, 747 $cm^{-1}$.

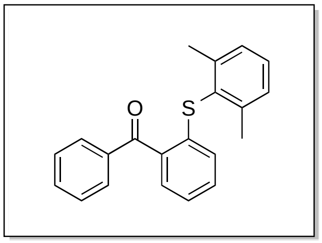

**2-(2,6-dimethylphenylthio)-benzophenone** According to General Procedure B, CuI (20.9 mg, 0.11 mmol), DMPHEN hemihydrate (21.7 mg, 0.10 mmol), 2-iodobenzophenone (305 mg, 0.990 mmol), 2,6-dimethylthiophenol (140 μL, 1.10 mmol), toluene (6 mL), and NaO*t*-Bu (143 mg, 1.5 mmmol) were heated for 20 h. The product was isolated as a white solid (170 mg, 0.534 mmol, 54% yield) by column chromatography ($SiO_2$, hexanes). $^1$H NMR (400 MHz, $CDCl_3$): δ 7.86 (d, *J* = 6.8 Hz, 2H), 7.61 (t, *J* = 7.4 Hz, 1H), 7.54 – 7.46 (m, 2H), 7.44 (d, *J* = 7.6 Hz, 1H), 7.25 – 7.06 (m, 5H), 6.68 (d, *J* = 8.0 Hz, 1H), 2.38 (s, 6H). $^{13}$C NMR (101 MHz, $CDCl_3$): δ 196.7, 144.0, 139.7, 137.7, 136.2, 133.1, 131.3, 131.0, 130.6, 130.3, 129.5, 128.6, 128.5, 126.3, 123.9, 21.9. LR-MS (EI) (*m*/*z*) calculated for $[C_{21}H_{18}OS]^+$: 318.1, found: 318.1. FT-IR (thin film): 3057, 2972, 2949, 2919, 1656 (C=O), 1597, 1580, 1462, 1432, 1315, 1284, 1254, 924, 762, 742, 699, 638 $cm^{-1}$.

## III. Molar Conductivity Measurements

Conductivity measurements were made using a VWR SB80PC sympHony Meter and conductivity probe. The meter was calibrated using aqueous NaCl solutions. All measurements were made using 1 mM solutions of analyte in acetonitrile at 21 °C and corrected to 25 °C using a linear correction of 2.1% per °C.

**Table S1.** Molar conductivities of measured compounds.

| **Compound** | **$\Lambda_m$ (S cm$^2$ mol$^{-1}$)** |
|---|---|
| Ferrocene | 0.45 |
| [TBA][$PF_6$] | 168.1 |
| **1** | 128.5 |

## IV. Spectroscopic Identification of Copper(II) Species

**Identification by UV-Vis spectroscopy.** Using propionitrile stock solutions to deliver each reagent, solutions of $[Cu^{I}(SAr)_2]Na$ (4. 6 mM, 1 mL, 4.6 μmol), PhI (49 mM, 0.5 mL, 24.5 μmol), and sodium 2,6-dimethylthiophenolate (24 mM, 1 mL, 24.0 μmol) were transferred to a cuvette in a nitrogen-filled glovebox. The reaction mixture was diluted with additional propionitrile (1.5 μM in $[Cu^{I}(SAr)_2]Na$), and the cuvette sealed with a Teflon valve and brought outside of the glovebox. The vessel was cooled to -78 °C and irradiated with a 100-W Hg lamp for 5 min, resulting in a blue solution. The cuvette was quickly transferred to the cooled UV-Vis cryostat (-80 °C) and its spectrum was collected. Control experiments were conducted identically but with exclusion of one or more components and dilution to a total volume of 3 mL.

**Identification by EPR spectroscopy.** The model complex $[Cu^{I}(SAr)_2]Na$ (7.0 mg, 0.010 mmol), sodium 2,6-dimethylthiophenolate (8.2 mg, 0.051 mmol), and iodobenzene (14.1 mg, 0.070 mmol) were diluted in 1:1 propionitrile:butyronitrile (2 mL). An aliquot of the solution was transferred to an EPR tube and sealed. Outside of the glovebox, the tube was irradiated with a 100-W Hg lamp for 5 min at -78 °C. The sample was immediately transferred to a liquid nitrogen-filled dewar and analyzed by X-band EPR spectroscopy. Control experiments were prepared identically but with exclusion of one or more components. Spin quantification was performed by comparing the double integral of the derivative spectrum of the photolyzed reaction mixture against that of a solution of various copper(II) sources of known concentration.

## V. Identification of 1 by ESI-MS

To a borosilicate tube in a nitrogen-filled glovebox was added, sequentially, CuI (6.3 mg, 0.033 mmol), NaO*t*-Bu (31.9 mg, 0.33 mmol), iodobenzene (37 µL, 0.33 mmol), and acetonitrile (1 mL). The vessel was fitted with a septum and removed from the glovebox. 2,6-dimethyl thiophenol (44 µL, 0.33 mmol) was added *via* syringe. The heterogeneous reaction mixture was stirred at 0 °C for 1 h under continuous illumination by a 100-W Hg lamp. An aliquot was drawn *via* a syringe equipped with a filter, and the sample diluted in acetonitrile. Subsequently, the sample was analyzed by ESI-MS.

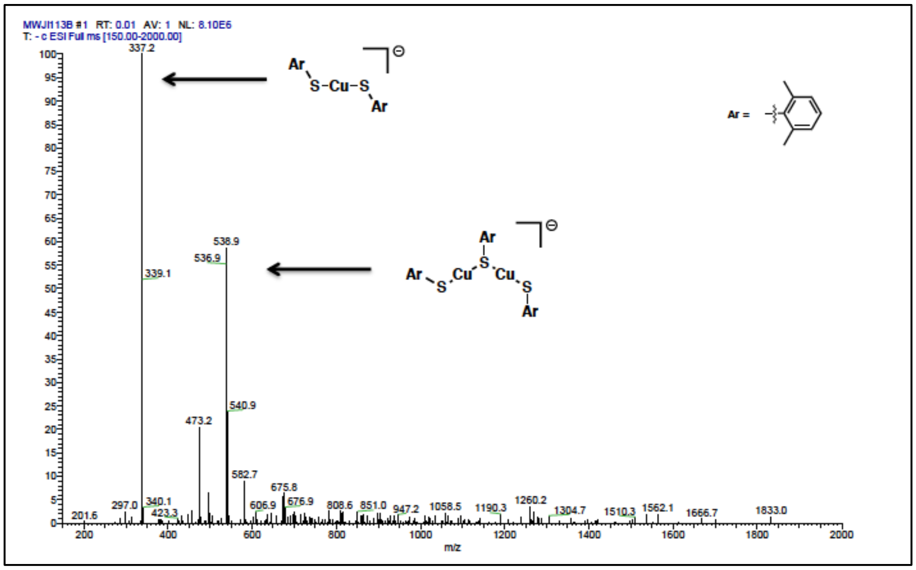


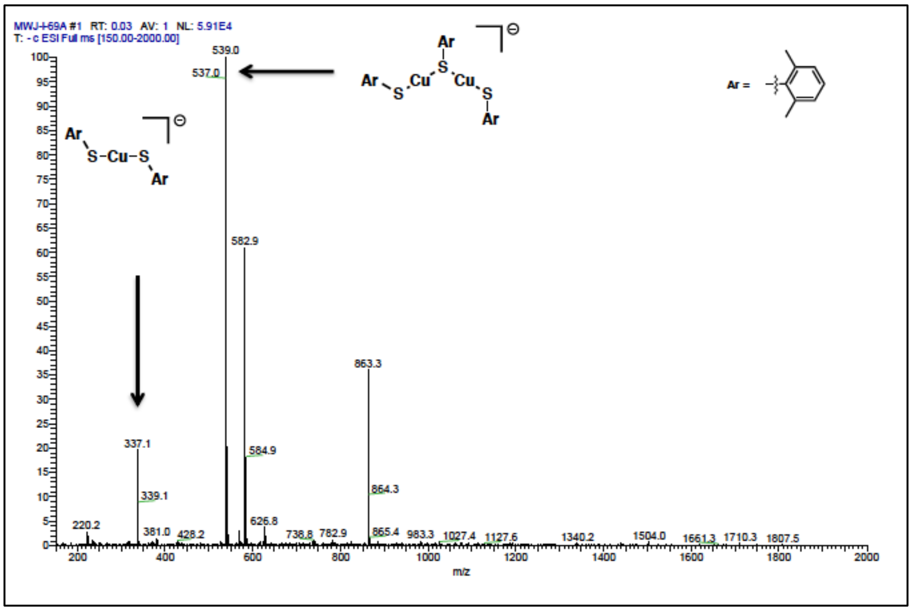


**Figure S2.** ESI-MS of **1**. Generated during catalysis (top) and independently synthesized (bottom).

# VI. Radical Clock Experiments

*All reaction mixtures were analyzed for coupled cyclized product, uncyclized coupled product, starting material, protodehalogenated starting material, and cyclized protodehalogenated product. Yields were determined by GC with the assistance of dodecane as an internal standard.*

**Stoichiometric Reaction of 1 with Radical Clocks.** In a nitrogen filled glovebox, a borosilicate test tube was charged with **1** (7.1 mg, 0.010 mmol), electrophile (0.010 mmol), and acetonitrile (0.02 M). The reaction mixture was photolyzed for 5 h at which time it was diluted with diethyl ether and dodecane was added (0.010 mmol). The mixture was filtered through silica and analyzed by GC.

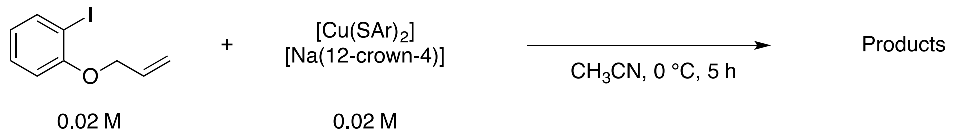


**Table S2.** Reactivity of **1** with 1-(allyloxy)-2-iodobenzene.

| | 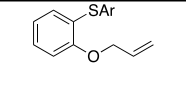 | 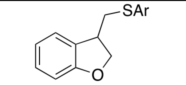 | 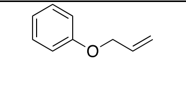 | 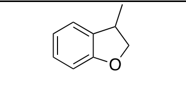 | 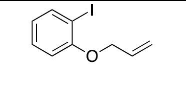 |
|---|---|---|---|---|---|
| Run 1 | 2% | 50% | 0% | 4% | 38% |
| Run 2 | 2% | 46% | 0% | 4% | 44% |

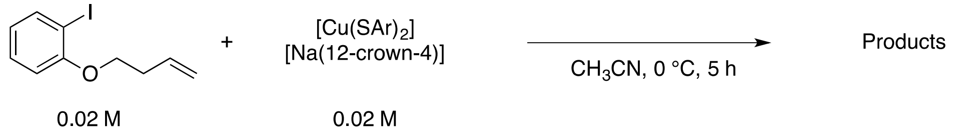


**Table S3.** Reactivity of **1** with 1-(but-3-en-1-yloxy)-2-iodobenzene.

| | 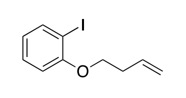 | 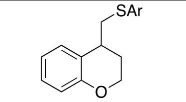 | 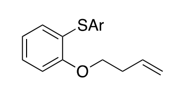 | 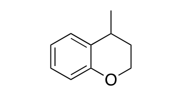 | 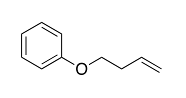 |
|---|---|---|---|---|---|
| Run 1 | 43% | 39% | 8% | - | - |
| Run 2 | 35% | 46% | 8% | - | - |

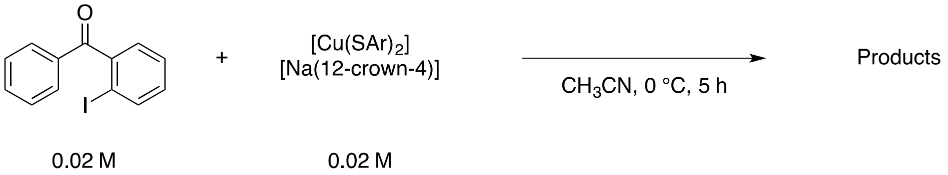


**Table S4.** Reactivity of **1** with 2-iodobenzophenone.

| | 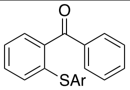 | 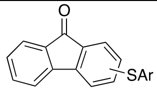 | 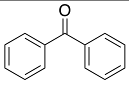 | 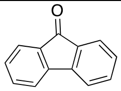 | 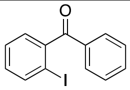 |
|---|---|---|---|---|---|
| Run 1 | 46 | 0% | 8% | 0% | 11% |
| Run 2 | 41 | 0% | 4% | 0% | 23% |

**Determination of Radical Clock Stability.** All radical clocks were subjected to the same conditions as in the stoichiometric reaction (*vide supra*) but in the absence of [Cu$^{I}$(SAr)$_2$]Na.

**Table S5.** Stability of radical clocks.

| Clock | Percent Recovery |
|---|---|
| 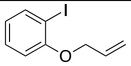 | 92 |
| 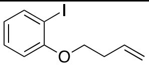 | 96 |
| 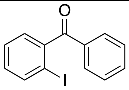 | 98 |

# VII. Reactivity of [Cu$^{I}$(SAr)$_2$]Na

*All reaction mixtures were analyzed for product, unreacted phenyl halide, biphenyl, and succinonitrile. Yields were determined by GC with the assistance of dodecane as an internal standard.*

### Stoichiometric Reaction of 1 with Phenyl Halides

In a nitrogen filled glovebox, a borosilicate test tube was charged with **1** (7.1 mg, 0.010 mmol), electrophile (0.010 mmol), and acetonitrile (0.02 M). The reaction mixture was photolyzed for 5 h at which time it was diluted with diethyl ether and dodecane was added (0.010 mmol). The mixture was filtered through silica and analyzed by GC.

**Table S6.** Reactivity of **1** with iodobenzene and control experiments.

| | Run 1 (% yeld) | Run 2 (% yield) |
|---|---|---|
| PhI | 54 | 57 |
| No light | 0 | 0 |
| No light or catalyst | 0 | 0 |

### Catalytic Reaction of Thiols and Aryl Halides under Homogeneous Conditions

*Model Reaction from Ref. 5*

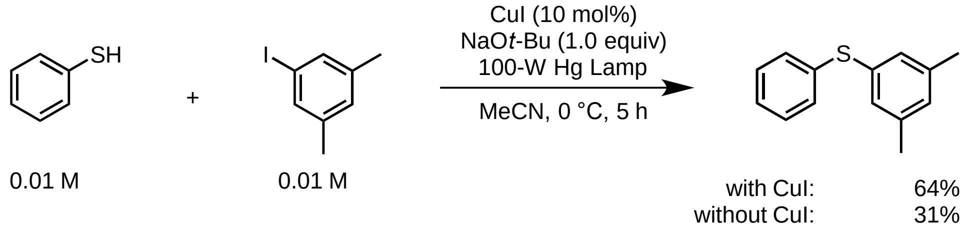


*Model Reaction for this Manuscript under Catalytic Conditions*

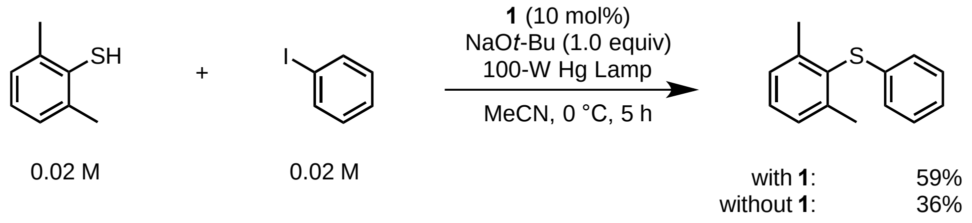

## VIII. Stern-Volmer Quenching Experiment

**Stern-Volmer Kinetic Analysis.** Complex **1** (30.1 mg, 0.0422 mmol) was diluted in acetonitrile (10 mL, 4.22 mM). Iodobenzene (618 mg, 3.03 mmol) was diluted in acetonitrile (10 mL, 303 mM). An acetonitrile solution of **1** (1.2 mM) was prepared with varying amounts of iodobenzene solution.

Data were analyzed using Matlab R2015A with the default curve fitting function.

**Table S7.** Excited-state lifetime as a function of quencher concentration.

| Concentration of PhI (mM) | Lifetime (μs) |
|---|---|
| 0 | 6.8 |
| 43.3 | 6.0 |
| 86.6 | 4.6 |
| 130 | 4.0 |
| 173 | 3.6 |

## IX. Steady-State Fluorimetry Experiment

**Emission and Excitation of 1.** A 25 μM solution of **1** in acetonitrile was excited using a Xe arc lamp (425 W) at 353 nm and the right angle emission detected at 675 nm. A 470 nm long-pass filter was used in determining both the excitation maximum and minimum.

## X. Reactivity of 1-(but-3-en-1-yloxy)-2-iodobenzene with $[Cu^{I}(SAr)_2]Na$

A stock solution of 1-(but-3-en-1-yloxy)-2-iodobenzene (1.0 mL, 0.010 M, 0.010 mmol) was added to a borosilicate tube containing $[Cu^{I}(SAr)_2]Na$. The reaction mixtures were photolyzed at 0 °C for 5 h with a mercury lamp. The reaction mixtures were then passed through a plug of silica diluted with ether, and the product distribution determined by GC.

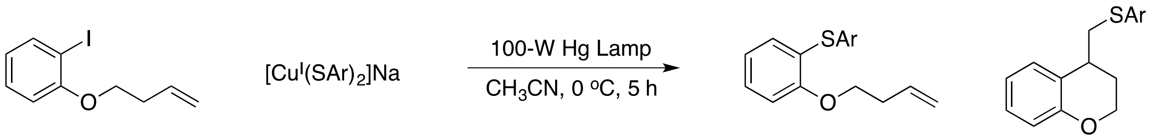


(0.010 M) X Y

**Table S8.** Product distribution in the reaction of **1** with 1-(but-3-en-1-yloxy)-2-iodobenzene

| $[Cu^{I}(SAr)_2]Na$ (mmol) | Yield X (%) | Yield Y (%) | Ratio |
|---|---|---|---|
| 1.0 | 6.8 | 31 | 4.6 |
| 1.5 | 9.3 | 39 | 4.2 |
| 2.0 | 5.9 | 23 | 3.9 |
| 2.5 | 9.6 | 44 | 4.6 |

## XI. VT-NMR Study of 1

The $^{1}H$ NMR spectrum of **1** in $CD_3CN$ (5 mM) was collected at 22 °C (bottom). The sampled was cooled to -30 °C in the probe and an additional spectrum collected.

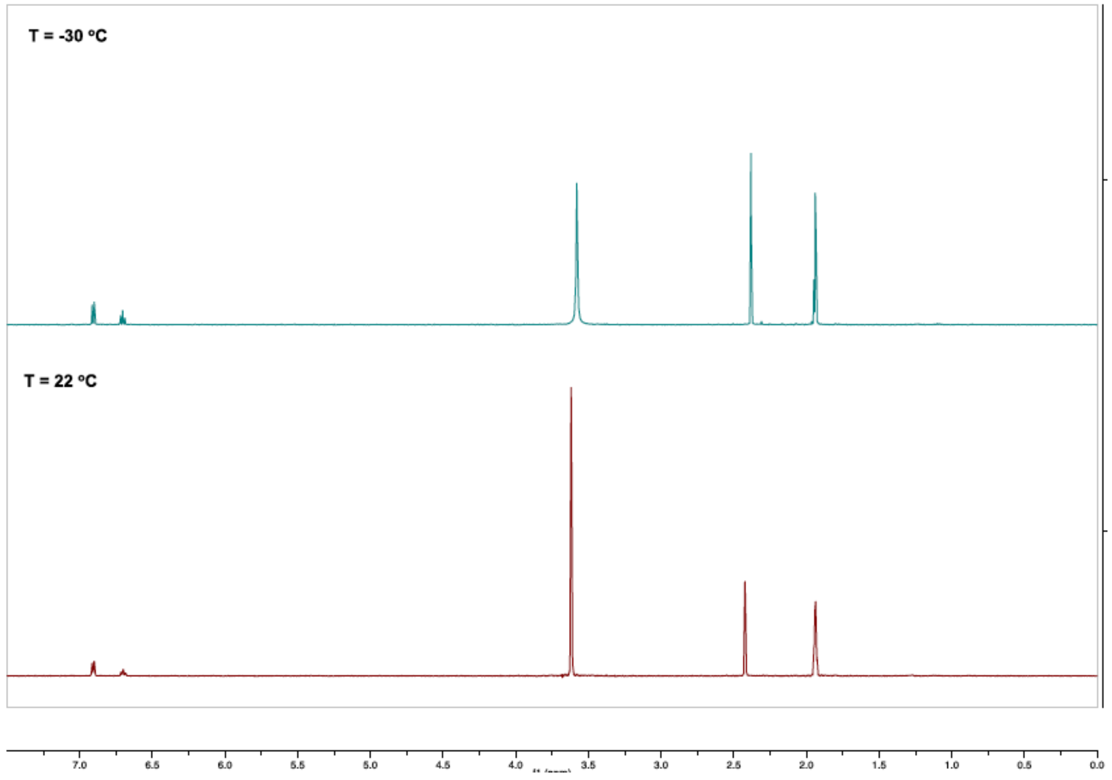


**Figure S3.** Low temperature (-30 °C, top) and ambient temperature (22 °C, bottom) $^{1}H$ NMR of **1**.

## XII. DOSY Experiment

**DOSY Procedure.** $[Cu^I(SAr)_2]Na$ (5 mg, 7 μmol) and decamethylferrocene (0.3 mg, 0.9 μmol) were weighed into an NMR tube, and $CD_3CN$ (0.5 mL) was added. A DOSY spectrum was acquired on a Varian 500 MHz spectrometer with a probe temperature of 25.0 °C, and the diffusion constants were calculated by exponential fit to the individual spectra. Hydrodynamic radii were calculated from the diffusion constants using the Stokes-Einstein equation.

**Table S9. Measured Hydrodynamic Radii.**

| δ (ppm) | Assignment | D ($10^{-10}$ m$^2$/s) | Hydrodynamic Radius (Å) |
|---|---|---|---|
| 1.71 | $Cp^*_2Fe$ | 17.22(7) | 3.70 |
| 1.98 | $CD_3CN$ | 39.2(6) | 1.62 |
| 2.47 | $[Cu(SAr)_2]^-$ | 14.58(4) | 4.19 |
| 3.66 | $[Na(12\text{-crown-}4)_2]^+$ | 15.23(6) | 4.39 |

**Calculation of Molar Volumes.** Molar volumes were calculated from the DFT-optimized geometries. In Gaussian 09, a single point calculation was run using the BP86 functional and def2-TZVP basis set for all atoms. The molar volume was then calculated by Monte-Carlo integration over the electron density grid ('Volume' keyword, 0.001 e-/Bohr$^3$ cutoff density, 1000 test points/Bohr$^3$). All volume calculations were run in triplicate due to the random error associated with Monte-Carlo methods.

**Table S10. DFT-Calculated Radii.**

| Species | Volume (1$^{st}$ run, cm$^3$/mol) | Volume (2$^{nd}$ run, cm$^3$/mol) | Volume (3$^{rd}$ run, cm$^3$/mol) | Average (cm$^3$/mol) | Radius (Å) |
|---|---|---|---|---|---|
| $[Na(12\text{-crown-}4)_2]^+$ | 280.4 | 286.0 | 281.6 | 282.7(23) | 4.82 |
| $[Cu(SAr)_2]^-$ | 236.9 | 238.8 | 242.7 | 239.5(21) | 4.55 |
| $[Cu_2(SAr)_3]^-$ | 364.2 | 359.3 | 361.8 | 361.8(14) | 5.25 |
| $[Cu_2(SAr)_4]^{2-}$ | 476.2 | 461.5 | 460.6 | 466.1(30) | 5.74 |
| $Cp^*_2Fe$ | 256.1 | 256.4 | 255.4 | 256.0(5) | 4.66 |

## XIII. Actinometric Studies

### Determination of light intensity:

All actinometric experiments were conducted in a Jobin Yvon Spec Fluorolog-3-11 fluorimeter with a 425 W Xe arc lamp using an excitation wavelength of 365 nm and an excitation slit width of 10 nm. The fluorimeter lamp was allowed to warm up for at least one hour prior to irradiation of samples. The photon flux of the fluorimeter was determined by ferrioxalate actinometry using the method of Bolton,[12] using a quantum yield of 1.28 for ferrioxalate reduction.[13] Solutions were irradiated for 0, 20, 40, and 60 seconds, and the quantum yield was determined for each sample. A photon flux of 2.9(3) * $10^{-8}$ einsteins $s^{-1}$ was calculated by averaging all time points.

Sample photon flux calculation for 20 second photolysis:

$$mol\ Fe(II) = \frac{V * 10 * A}{1000 * \varepsilon * V_1} = \frac{3.0\ mL * 10 * 0.28}{1.0\ mL * 1000 * \ 11{,}100\ L\ mol^{-1}\ cm^{-1}} = 7.5 * 10^{-7}\ mol$$

$$photon\ flux = \frac{mol\ Fe(II)}{Phi * t * f} = \frac{7.5 * 10^{-7}}{1.28 * 20} = 2.9 * 10^{-8}\ einsteins\ s^{-1}$$

Where $V$ is the volume irradiated, $V_1$ is the aliquot volume, and ε is the extinction coefficient of the Fe(II) phenanthroline complex.

### Determination of quantum yield for stoichiometric model reaction:

To a 1-cm cuvette with a Kontes valve or screw cap was added $[Cu^{I}(SAr)_2]Na$ (0.06 mmol, 1 equiv), PhI (0.06 mmol, 1 equiv), and MeCN (3.0 mL). A stir bar was added, and the cuvette was sealed. The absorbance of the solution at 365 nm was measured by UV-Vis prior to irradiation. The sample was cooled to 0 °C and placed in a cuvette holder and cooled to 0 °C with an internal cooling loop. While stirring, the sample was irradiated for one hour. The absorbance of the solution at 365 nm was measured by UV-Vis following irradiation. After irradiation, $Et_2O$ (3 mL) and dodecane (0.06 mmol, 1 equiv) were added. The resulting mixture was passed through a silica plug and was analyzed by GC.

The fraction of light absorbed by the solutions at 365 nm was determined by taking the average between the absorbance prior to irradiation and post irradiation. This was converted to fraction of light absorbed (*f*), where *A* is the average absorbance.

$$f = 1 - 10^{-A}$$

The quantum yield of the reaction was then determined using the following equation:

$$\Phi = \frac{mol\ Fe(II)}{photon\ flux * t * f}$$

The reported quantum yield of 0.08 is the average of two experiments that gave quantum yields of 0.079 and 0.074.

## XIV. Emission Spectrum of 100-W Hg Lamp

*The emission spectrum was measured using a J & M Analytik TIDAS S 300 K detector.*

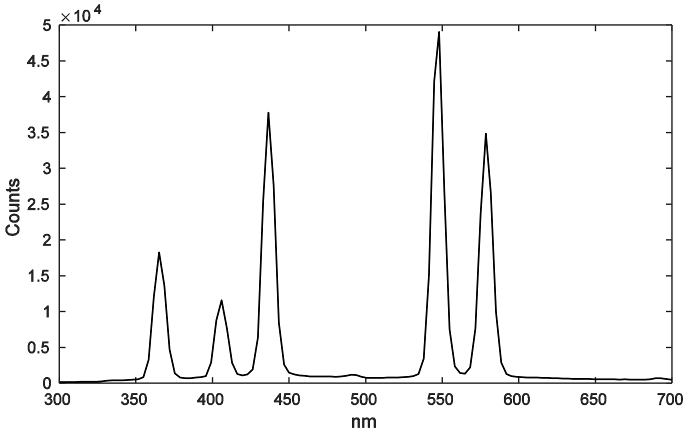


**Figure S4.** Emission spectrum of 100-W Blak-Ray Long Wave Ultraviolet Lamp (Hg)

## XV. Determination of Molar Absorptivities (ε)

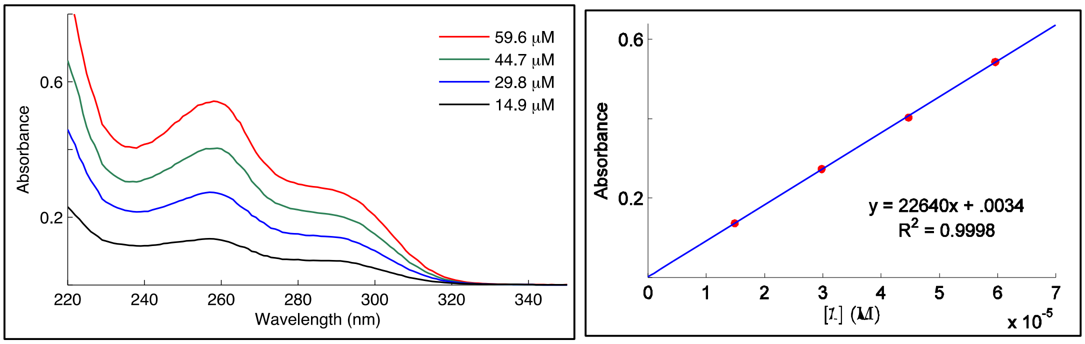


**Figure S5.** Absorbance spectra of $[Cu^{I}(SAr)_2]Na$ in acetonitrile at various concentrations (left). Absorbance at 258 nm as a function of concentration (right).

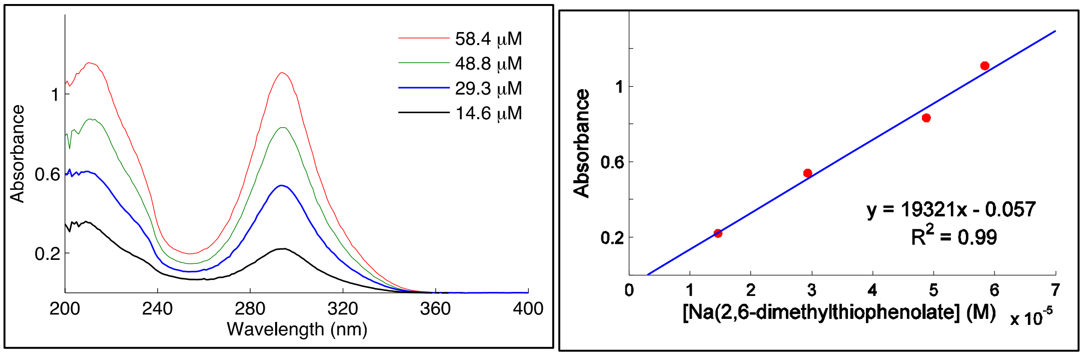


**Figure S6.** Absorbance spectra of sodium2,6-dimethylthiophenolate in acetonitrile at various concentrations (left). Absorbance at 292 nm as a function of concentration (right).

## XVI. Absorption Spectra of 1 in the Presence of 2

### UV-Vis Spectra of [Cu$^{I}$(SAr)$_2$]Na in Varying Concentrations of Sodium 2,6-Dimethylthiophenolate

A propionitrile solution of [Cu$^{I}$(SAr)$_2$]Na (30 μM, 2.5 mL) was added to a septum-sealed cuvette and its spectrum collected. A propionitrile solution of **2** (8.1 mM) was added in 20-μL volumes *via* syringe to the cuvette and the spectrum of the resulting solution collected.

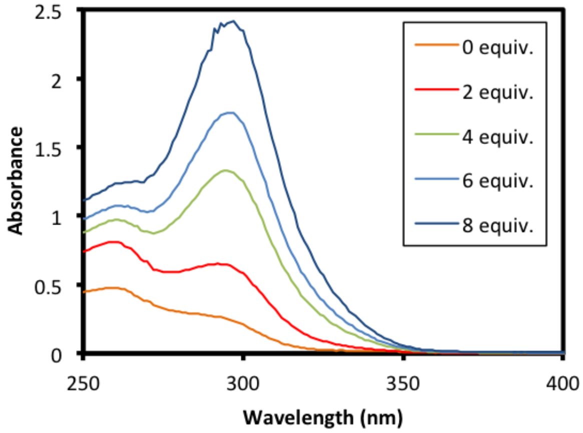


**Figure S7.** Optical spectra of **1** in the presence of increasing concentrations of sodium 2,6-dimethylthiophenolate.

### UV-Vis Spectra of [Cu$^{I}$(SAr)$_2$]Na at Various Temperatures in the Presence of Sodium 2,6-Dimethylthiophenolate

A propionitrile solution of [Cu$^{I}$(SAr)$_2$]Na (30 μM) in the presence of sodium 2,6-dimethythiophenolate (80 μM) was cooled from 25 to -80 °C and the spectrum collected in 20-degree intervals.

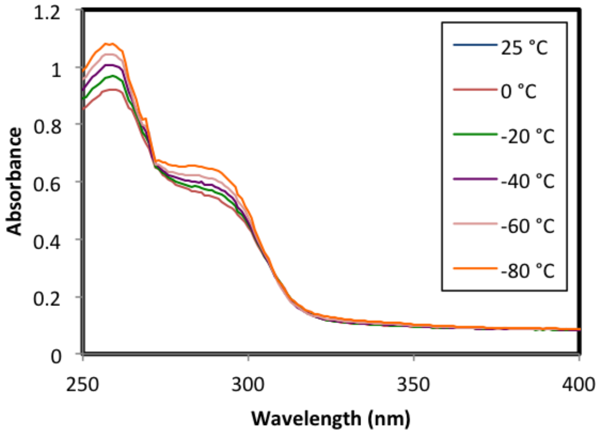


**Figure S8.** Optical spectra of **1** in the presence of sodium 2,6-dimethylthiophenolate at variable temperature.

# XVII. DFT Calculations

**General Considerations.** The Orca 3.0.1 program was used for all calculations.[14] All optimizations and energy calculations were conducted with tight convergence criteria using the BP86 functional[15,16] and def2-TZVP basis set, with the def2-TZVP effective core potential used for iodine.[17] Open and closed shell species were modeled within the unrestricted and restricted Kohn-Sham formalisms, respectively. When energies were compared between open- and closed-shell species, the unrestricted Kohn-Sham formalism was used for all species. All geometry optimizations were conducted without symmetry constraints using gradient methods. Ground state geometries were verified as true minima by the absence of imaginary frequencies. All energies reported are Gibbs free energies at 298.15 K and include translational, rotational, vibrational, and solvation energy contributions. Solvation was treated with the conductor-like screening model, using default parameters for acetonitrile in all cases.

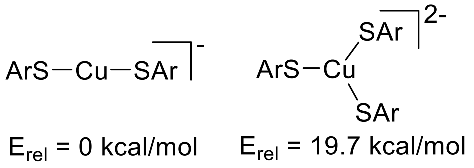


**Figure S9**. Calculated free energies of two possible Cu(I) speciations.

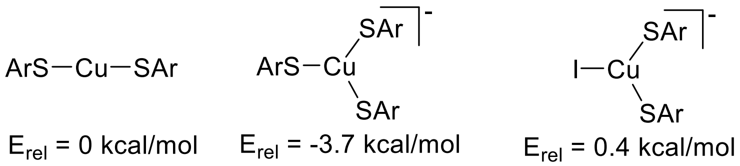


**Figure S10**. Calculated free energies of three possible Cu(II) speciations.

**Table S11.** Free energies of computed molecules.

| Compound | Gibbs Free Energy (Hartrees) |
|---|---|
| [2,6-dimethylthiophenolate]$^-$ | -708.6642 |
| [Cu(2,6-dimethylthiophenolate)$_2$]$^-$ | -3058.0630 |
| [Cu(2,6-dimethylthiophenolate)$_3$]$^{2-}$ | -3766.6958 |
| Cu(2,6-dimethylthiophenolate)$_2$ | -3057.8949 |
| [Cu(2,6-dimethylthiophenolate)$_3$]$^-$ | -3766.5650 |
| [Cu(2,6-dimethylthiophenolate)$_2$I]$^-$ | 3355.9502 |
| I$^-$ | -298.0560 |

**EPR Parameter Simulation.** DFT calculations of the EPR parameters were conducted using the BP86 functional, the CP(PPP) basis set[18] for copper, and the def2-TZVP basis set for all other atoms. Integration was performed over a Lebedev grid with 770 points (Grid7) for copper and 590 points (Grid 6) for all other atoms. TD-DFT calculations were conducted using the Tamm-Dancoff approximation.[19]

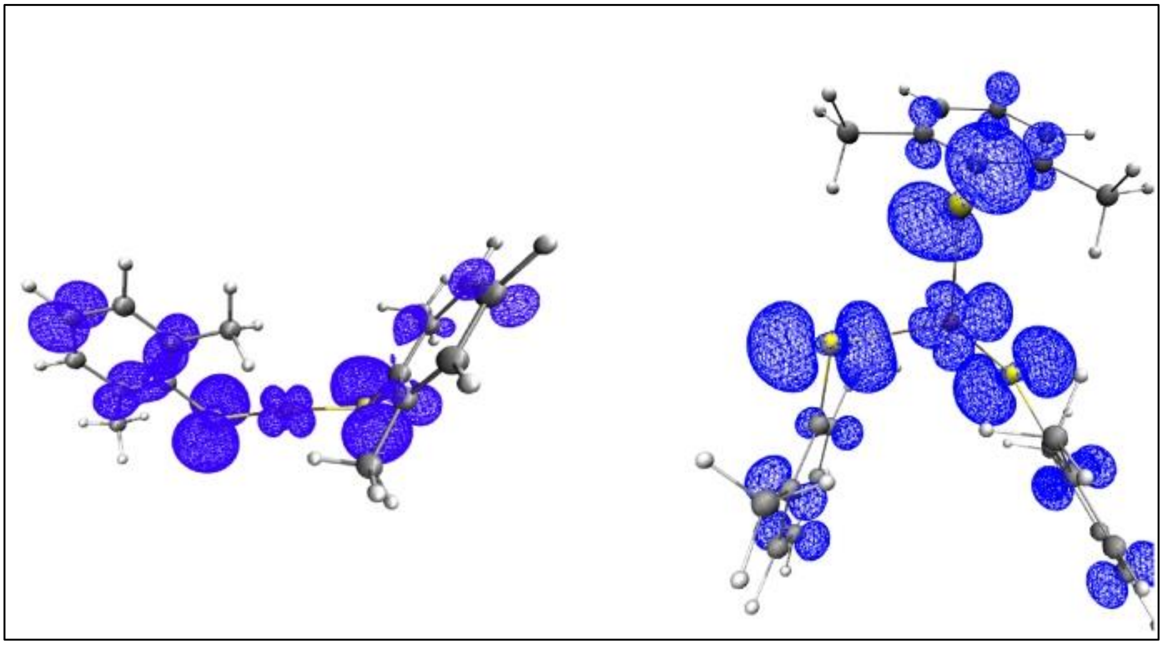

**Figure S11.** Spin density plots of Cu(2,6-dimethylthiophenolate)$_2$ (left) and [Cu(2,6-dimethylthiophenolate)$_3$]$^-$ (right).

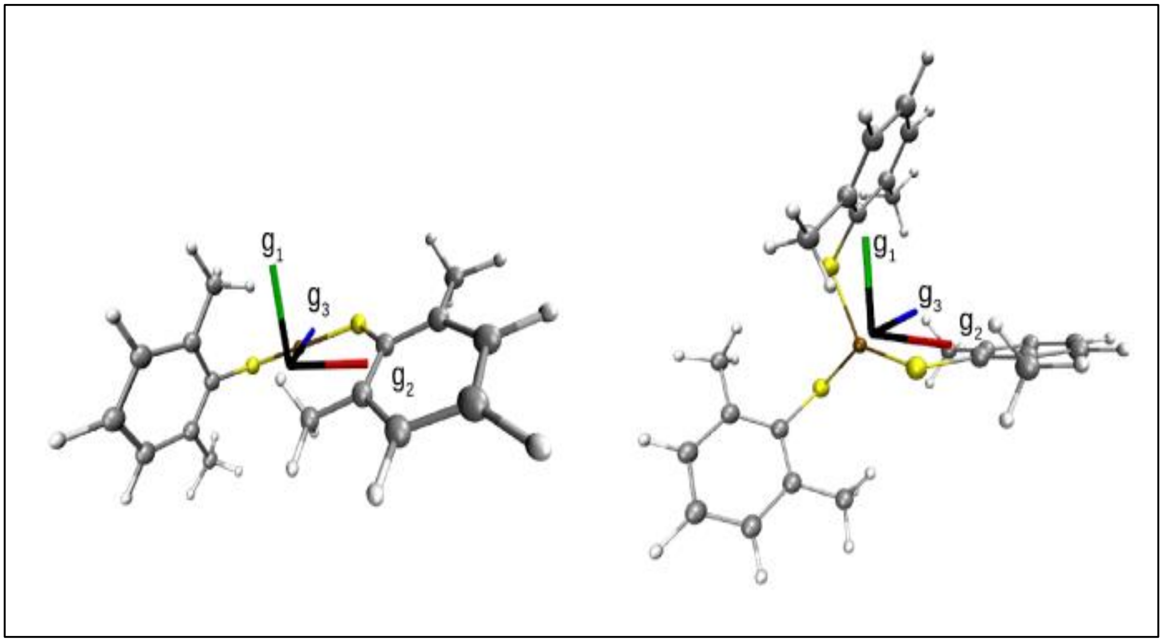


**Figure S12.** DFT structures of Cu(2,6-dimethylthiophenolate)$_2$ (left) and [Cu(2,6-dimethylthiophenolate)$_3$]$^-$ (right) showing the orientation of the *g* tensor.

## XVIII. Probe of Direct Coupling between [$Cu^{I}(SAr)_2$]Na (1) and Aryl Radical

**Monitoring of 1 and 6 at -20 °C.** In the glovebox**, 1** (7.2 mg, 0.010 mmol, 1.0 equiv) and **6** (2.2 mg, 0.010 mmol, 1.0 equiv) were weighed into a J. Young NMR tube. The tube was sealed and 1.0 mL $CD_3CN$ was added by vacuum transfer. The tube was thawed to -20 °C and mixed by gently stirring for 5 minutes before refreezing. The frozen tube was then transferred to an NMR spectrometer pre-cooled to -20 °C and the reaction was monitored by $^1$H NMR spectroscopy over 30 minutes, showing minimal (<2%) formation of **7**.

**Reaction of 1 and 6.** In the glovebox**, 1** (7.2 mg, 0.010 mmol, 1.0 equiv) and **6** (2.2 mg, 0.010 mmol, 1.0 equiv) were weighed into a 4-mL septum-capped vial with a stir bar and acetonitrile (1.0 mL) was added *via* syringe. The reaction was then stirred for 30 minutes at ambient temperature. After stirring for 30 minutes, dodecane (4.5 µL, 0.020 mmol, 2.0 equiv) and 1 mL diethyl ether were added, and the reaction mixtures were filtered through a short plug of silica and analyzed by GC. Yield of **7** = 57% (average of three experiments).

**Reaction of 1 and 6 with $Cp^*_2Fe$.** In the glovebox**, 1** (7.2 mg, 0.010 mmol, 1.0 equiv), **6** (2.2 mg, 0.010 mmol, 1.0 equiv), and decamethylferrocene (3.5 mg, 0.011 mmol, 1.1 equiv) were weighed into a 4-mL septum-capped vial with a stir bar. The vial was cooled to -20 °C and acetonitrile (1.0 mL) at -20 °C was added *via* syringe. The reaction was then stirred for 30 minutes at -20 °C and warmed to ambient temperature. After warming for 30 minutes, dodecane (4.5 µL, 0.020 mmol, 2.0 equiv) and 1 mL diethyl ether were added, and the reaction mixtures were filtered through a short plug of silica and analyzed by GC. Yield of **7** = 22% (average of two experiments).

**Monitoring of 1 and 6 with $Cp^*_2Fe$ at -20 °C.** In the glovebox**, 1** (7.2 mg, 0.010 mmol, 1.0 equiv), **6** (2.2 mg, 0.010 mmol, 1.0 equiv), and decamethylferrocene (3.5 mg, 0.011 mmol, 1.1 equiv) were weighed into a J. Young NMR tube. The tube was sealed and 1.0 mL $CD_3CN$ was added by vacuum transfer. The tube was thawed to -20 °C and mixed by gently stirring for 5 minutes before refreezing. The frozen tube was then transferred to an NMR spectrometer pre-cooled to -20 °C. $^1$H NMR measurements taken 5 minutes after insertion of the NMR tube into the spectrometer show complete consumption of **6**, demonstrating that the reaction between **6** and $Cp^*_2Fe$ does in fact occur at -20 °C and not upon warming.

**Reaction of 1 and 6 with [$Cp^*_2Fe$][$BF_4$].** In the glovebox**, 1** (7.2 mg, 0.010 mmol, 1.0 equiv), **6** (2.2 mg, 0.010 mmol, 1.0 equiv), and decamethylferrocenium tetrafluoroborate (4.1 mg, 0.010 mmol, 1.0 equiv) were weighed into a 4-mL septum-capped vial with a stir bar. The vial was cooled to -20 °C and acetonitrile (1.0 mL) at -20 °C was added *via* syringe. The reaction was then stirred for 30 minutes at -20 °C

and warmed to ambient temperature. After warming for 30 minutes, dodecane (4.5 μL, 0.020 mmol, 2.0 equiv) and 1 mL diethyl ether were added, and the reaction mixtures were filtered through a short plug of silica and analyzed by GC. Yield of **7** = 56% (average of two experiments).

**Reaction of 1 and 2-(allyloxy)benzenediazonium tetrafluoroborate.** In the glovebox**, 1** (7.2 mg, 0.010 mmol, 1.0 equiv) and 2-(allyloxy)benzenediazonium tetrafluoroborate (2.5 mg, 0.010 mmol, 1.0 equiv) were weighed into a 4-mL septum-capped vial with a stir bar. The vial was cooled to -20 °C and acetonitrile (1.0 mL) at -20 °C was added *via* syringe. The reaction was then stirred for 30 minutes at -20 °C. After stirring for 30 minutes, dodecane (4.5 μL, 0.020 mmol, 2.0 equiv) and 1 mL diethyl ether were added, and the reaction mixtures were filtered through a short plug of silica and analyzed by GC. Yield of 3-(2,6-dimethylphenylthiomethyl)-2,3-dihydrobenzo-furan = 99% (average of two experiments).

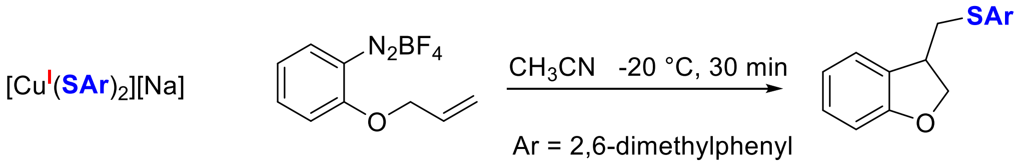


**Probe for Redox Equilibrium between 1 and [Cp*$_2$Fe][BF$_4$] at -20 °C.**

To probe the possibility of a redox equilibrium between **1** and [FeCp*$_2$][BF$_4$], we combined **1** with [FeCp*$_2$][BF$_4$] at –20 °C in $CH_3CN$ and observed ~20% consumption of [FeCp*$_2$][BF$_4$] over 30 minutes, consistent with a possible redox equilibrium between **1** and [FeCp*$_2$][BF$_4$] generating a thermally unstable Cu(II)-thiolate. To probe this possibility further, we employed TEMPOH as a trap for any generated Cu(II)-thiolate (TEMPOH = 1-hydroxyl-2,2,6,6-tetramethylpiperidine). Reaction of **1** with [FeCp*$_2$][BF$_4$] and TEMPOH at –20 °C in $CH_3CN$ resulted in complete consumption of [FeCp*$_2$][BF$_4$] within 10 minutes, not requiring but certainly consistent with the notion of a redox equilibrium between [FeCp*$_2$]$^+$ and **1**.

## XIX. Cage Escape Efficiency as a Function of Temperature

The rate of diffusional cage escape ($k_d$) can be given by the Eigen equation:[20]

$$k_d = \frac{2kT}{\pi\eta r^3}\left(\frac{b}{r}\right)\left(\frac{1}{1-e^{\frac{-b}{r}}}\right)$$

If one or both of the photogenerated radicals are uncharged, then the coulombic interaction between them can be taken to be zero, giving a simplified form of the Eigen equation. An analytical formula for viscosity in terms of three constants (A, B, and C) can then be substituted in:[21]

$$lim_{b\to 0}k_d = lim_{b\to 0}\frac{2kT}{\pi\eta r^3}\left(\frac{b}{r}\right)\left(\frac{1}{1-e^{\frac{-b}{r}}}\right) = \frac{2kT}{\pi\eta r^3} = \frac{2kT}{\pi r^3\cdot(10^3\cdot 10^{A+\frac{B}{C-T}})}$$

Assuming that the coupling rate follows the Eyring equation, the rate of in-cage recombination ($k_r$) is given by:

$$k_r = \frac{kT}{h}e^{\frac{-\Delta G^{\ddagger}}{RT}}$$

Therefore, the efficiency of cage escape ($\eta_{ce}$) is given by:

$$\eta_{ce} = \frac{k_d}{k_d+k_r} = \frac{\frac{2kT}{\pi r^3\cdot(10^3\cdot 10^{A+\frac{B}{C-T}})}}{\frac{2kT}{\pi r^3\cdot(10^3\cdot 10^{A+\frac{B}{C-T}})}+\frac{kT}{h}e^{\frac{-\Delta G^{\ddagger}}{RT}}} = \frac{1}{1+\frac{\pi r^3}{2h}\cdot(10^3\cdot 10^{A+\frac{B}{C-T}})\cdot e^{\frac{-\Delta G^{\ddagger}}{RT}}}$$

This function shows that the efficiency of cage escape should increase with decreasing temperature as long as there is a non-negligible barrier to coupling. Shown below is a representative plot of cage escape efficiency using a cage radius of 1 nm as has been approximated for other systems,[20] an arbitrary modest coupling barrier of 6 kcal/mol, and the viscosity constants for propionitrile (A = -4.8, B = -424.6, C = 3.8, Figure S11).

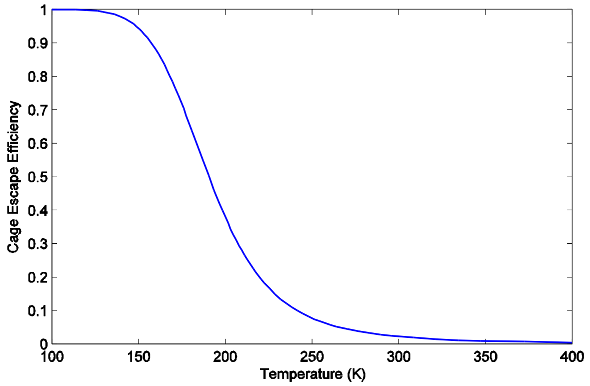


**Figure S13.** Representative plot of cage escape efficiency as a function of temperature, showing a significant increase in cage escape probability as the temperature is lowered.

## XX. Calculation of Excited State Reduction Potential of 1

The reduction potential of an excited state species can be calculated with the following equation:

$$\Delta G° = E°_{D^+/D} - E°_{A/A^-} - E_D^* - C$$

$E°_{D^+/D}$ : Reduction potential of donor

$E°_{A/A^-}$ : Reduction potential of acceptor

$E_D^*$ : Excited-state potential of donor

$C$ : Coulombic term

Assuming that $C$ is negligible in acetonitrile and that no acceptor is present, the equation is simplified:

$$\Delta G° = E°_{D^+/D} - E_D^*$$

Assuming that driving force is equal to the difference of the ground state potential of **1** and $E^{00}$ for **1***:

$$\Delta G° = -0.18 \text{ V} - (-2.4 \text{ V})$$

$$\Delta G° \approx -2.6 \text{ V}$$

## XXI. References


(1) Tsuda, T.; Yazawa, T.; Watanabe, K.; Fujii, T.; Saegusa, T. *J. Org. Chem.* **1981**, *46*, 192, g. C

(2) Jiang, H.; Bak, J. R.; Lopez-Delgado, F. J.; Jorgensen, K. A. *Green Chem.* **2013**, *15*, 3355Chem.p

(3) Carril, M.; SanMartin, R.; Dominguez, E.; Tellitu, I. *Green Chem.* **2007**, *9*, 315 Chem

(4) Dahlen, A.; Petersson, A.; Hilmersson, G. *Org. Biomol. Chem.* **2003**, *1*, 2423. Chem

(5) Uyeda, C.; Tan, Y.; Fu, G. C.; Peters, J. C. *J. Am. Chem. Soc.* **2013**, *135*, 9548 Chem.

(6) Erb, W.; Hellal, A; Albini, M.; Rouden, J.; Blanchet, J. *Chem. Eur. J.* **2014**, *20*, 6608-6612.

(7) LeSuer, R. J.; Buttolph, C.; Geiger, W. E. *Anal. Chem.* **2004**, *76*, 6395Chem.r

(8) Giffin, N. A.; Makramalla, M.; Hendsbee, A. D.; Robertson, K. N.; Sherren, C.; Pye, C. C.; Masuda, J. D.; Clyburne, J. A. C. *Org. Biomol. Chem.* **2011**, *9*, 3672. Chem

(9) Kim, H.; Lee, C. *Angew. Chem. Int. Ed.* **2012**, *51*, 12303Int. Ed

(10) Stoll, S.; Schweiger, A. *J. Magn. Reson.* **2006**, *178*, 42n.n.

(11) Bates, C. G.; Gujadhur, R. K.; Venkataraman, D. *Org. Lett.* **2002**, *4*, 2803ett.an

(12) Bolton, J. R.; Stefan, M. I.; Shaw, P.-S.; Lykke, K. R. *J. Photochem. Photobiol. Chem.* **2011**, *222*, 166m.ol.

(13) Demas, J. N.; Bowman, W. D.; Zalewski, E. F.; Velapoldi, R. A. *J. Phys. Chem.* **1981**, *85*, 2766–2771.

(14) Neese, F. *Wiley Interdiscip. Rev. Comput. Mol. Sci.* **2012**, *2*, 73l. S

(15) Perdew, J. P. *Phys. Rev. B* **1986**, *33*, 8822Rev. B

(16) Becke, A. D. *Phys. Rev. A* **1988**, *38*, 3098Rev. A

(17) Weigend, F.; Furche, F.; Ahlrichs, R. *J. Chem. Phys.* **2003**, *119*, 12753. Phys.

(18) Neese, F. *Inorg. Chim. Acta* **2002** *337*, 181-192.

(19) Hirata, S.; Head-Gordon, M. *Chem. Phys. Lett.* **1999**, *314*, 291 Phys.

(20) Olmsted, J.; Meyer, T. J. *J. Phys. Chem.* **1987**, *91*, 1649-1655.

(21) Viswanath, D. S.; Ghosh, T. K.; Prasad, D. H. L.; Dutt, N. V. K; Rani, K. Y. *Viscosity of Liquids*; Springer: Dordrecht, The Netherlands, 2007; pp. 443, 589.

## XXII. X-Ray Crystallographic Data

**Table S12.** Crystal Data and Structure Refinement for 1

| Identification Code | **1** |
|---|---|
| Empirical Formula | $C_{32}H_{50}CuNaO_8S_2$ |
| Formula Weight | 713.37 |
| Temperature/K | 100 |
| Crystal System | monoclinic |
| Space Group | $P2_1/c$ |
| a/Å | 10.5883(14) |
| b/Å | 21.967(2) |
| c/Å | 14.4286(16) |
| α/° | 90 |
| β/° | 95.682(6) |
| γ/° | 90 |
| Volume/Å$^3$ | 3339.6(7) |
| Z | 4 |
| $\rho_{calc}$ mg/mm3 | 1.419 |
| F(000) | 1512 |
| Crystal Size/mm$^3$ | 0.16 x 0.15 x 0.09 |
| Radiation | Mo Kα (λ = 0.71073) |
| 2Θ range for data collection | 4.6 to 74.0 |
| Indices Ranges | $-17 \leq h \leq 17$, $-37 \leq k \leq 36$, $-24 \leq l \leq 24$ |
| Reflections Collected | 141854 |
| Independent Reflections | 16419 ($R_{int}$ = 0.1249) |
| Data/Restraints/Parameters | 16419/0/401 |
| Goodness-of-fit on $F^2$ | 1.036 |
| Final R indices [I>2σ (I)] | $R_1$ = 0.0673, $wR_2$ = 0.0931 |
| Final R Indices [all data] | $R_1$ = 0.1542, $wR_2$ = 0.1114 |
| Largest diff. Peak/hole /eÅ$^{-3}$ | 0.640 / -0.547 |

# XXIII. $^{1}H$ and $^{13}C$ NMR Data

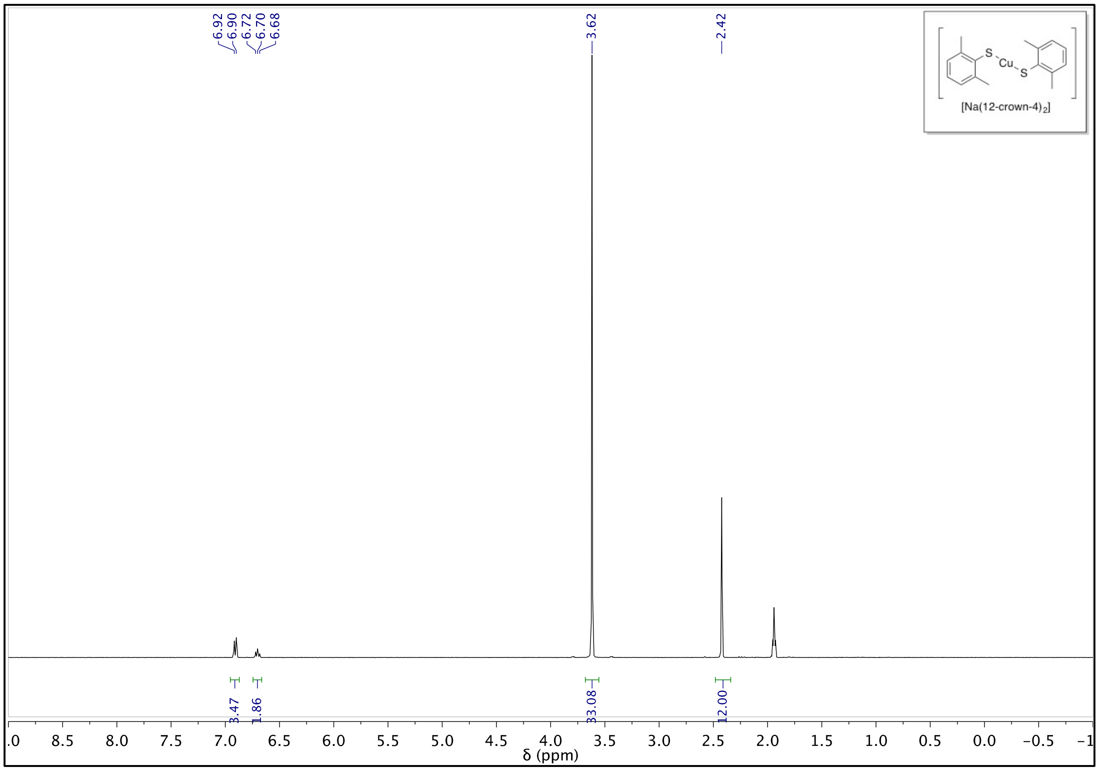


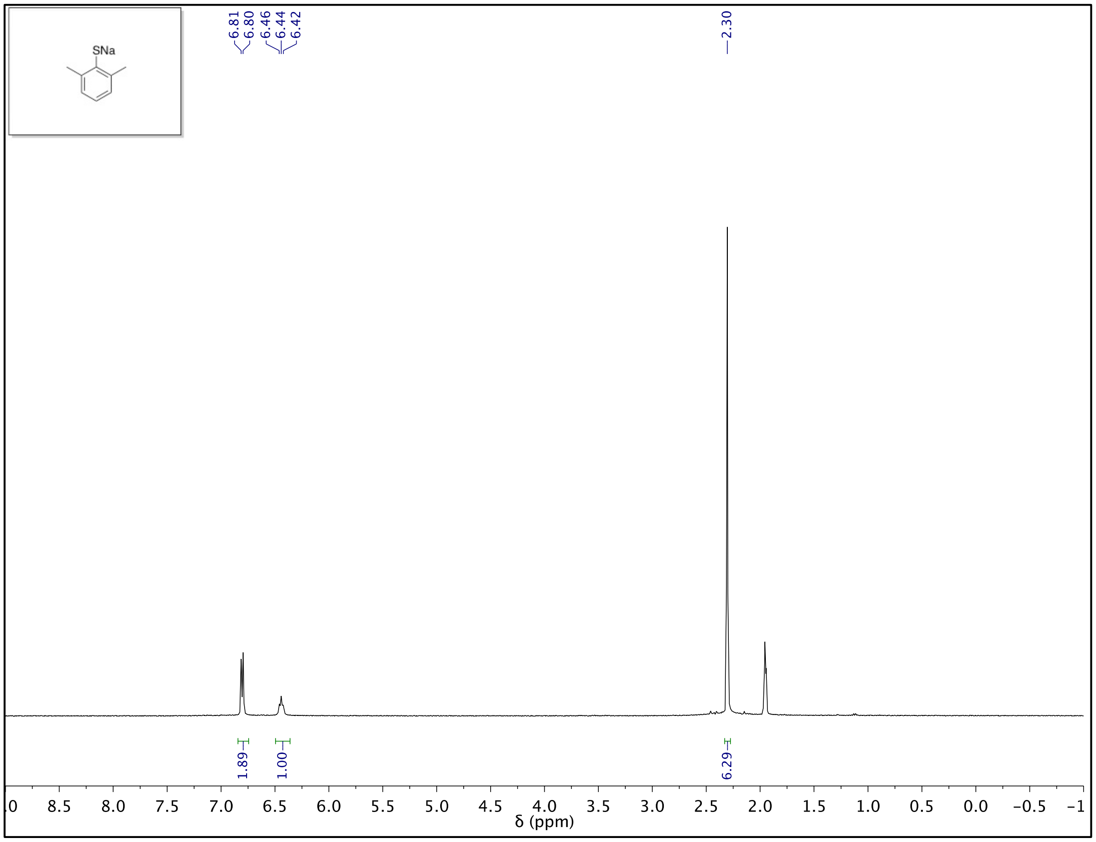

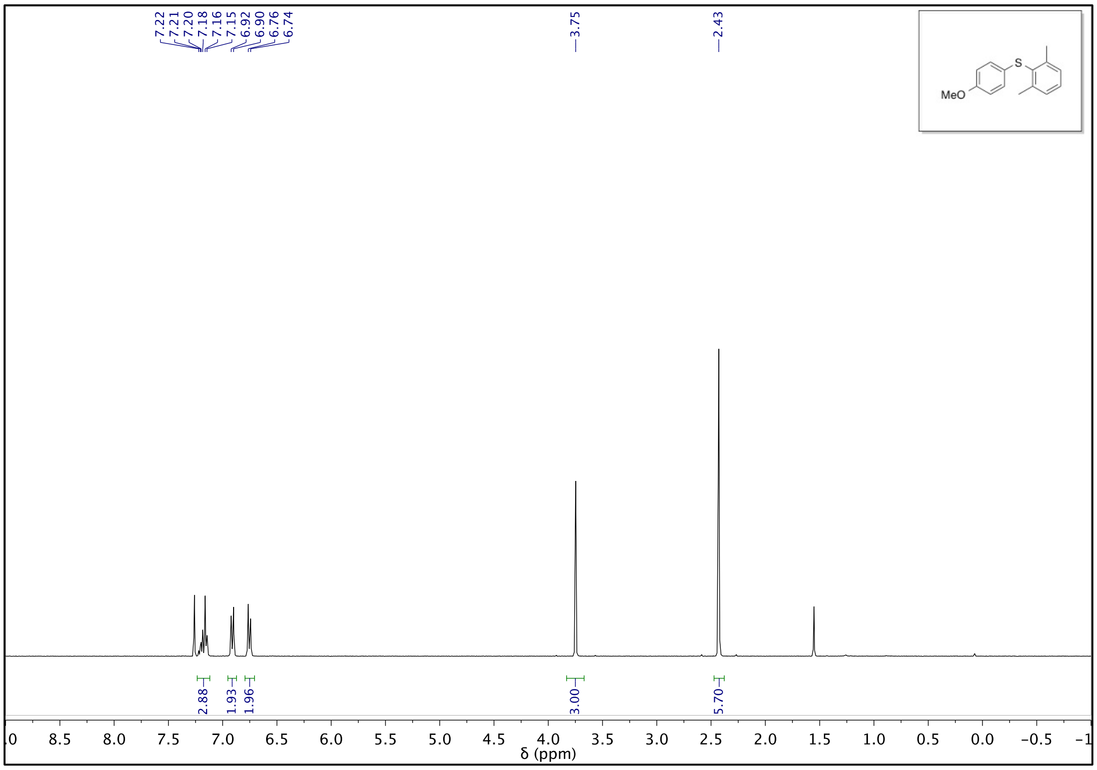
7.22
7.21
7.20
7.18
7.16
7.15
6.92
6.90
6.76
6.74
3.75
2.43
MeO
S
2.88
1.93
1.96
3.00
5.70
δ (ppm)

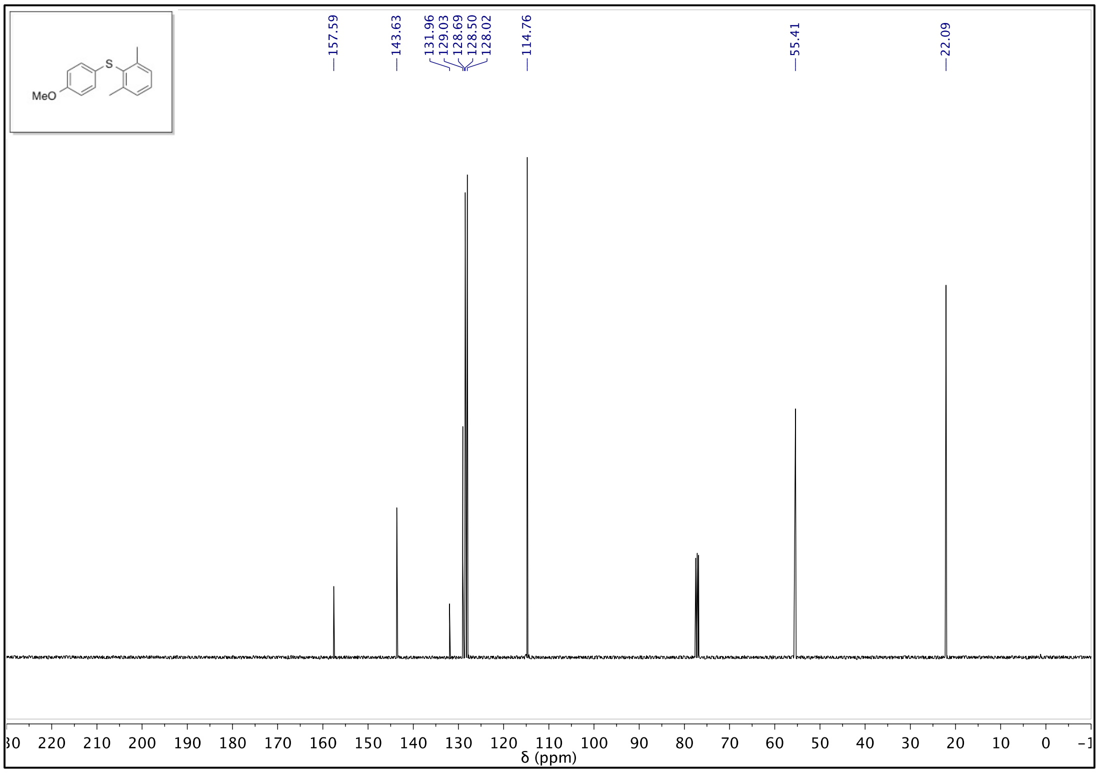
157.59
143.63
131.96
129.03
128.69
128.50
128.02
114.76
55.41
22.09
MeO
S
δ (ppm)

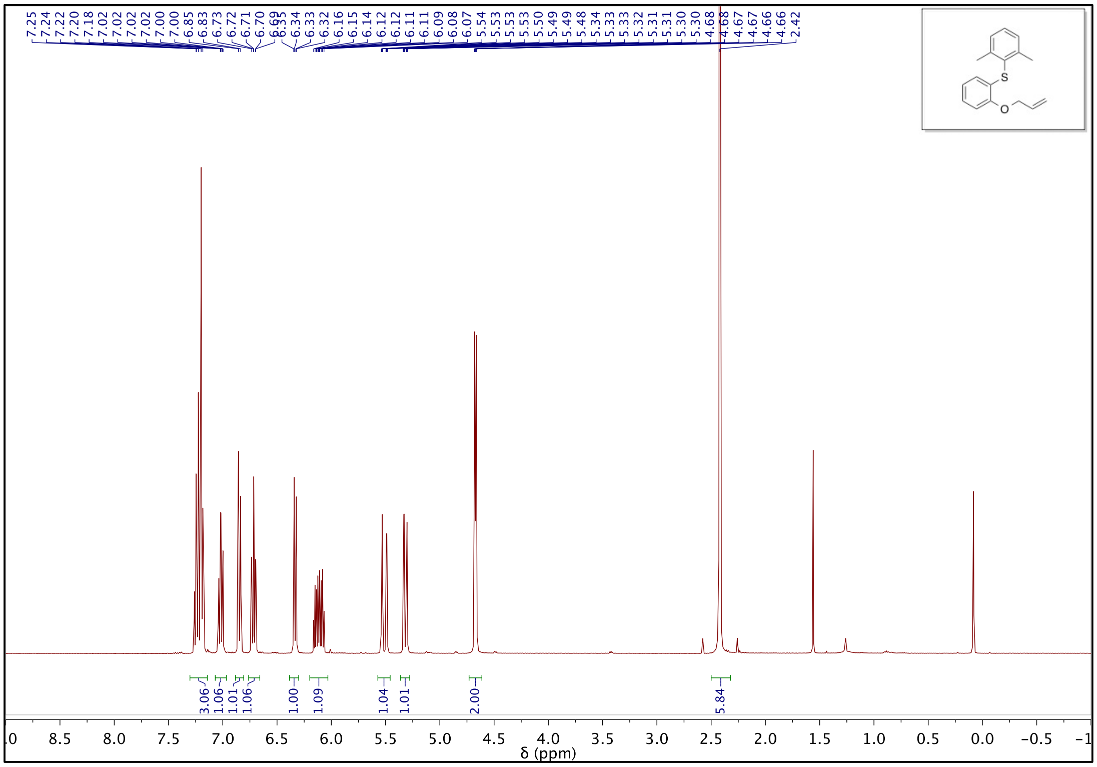

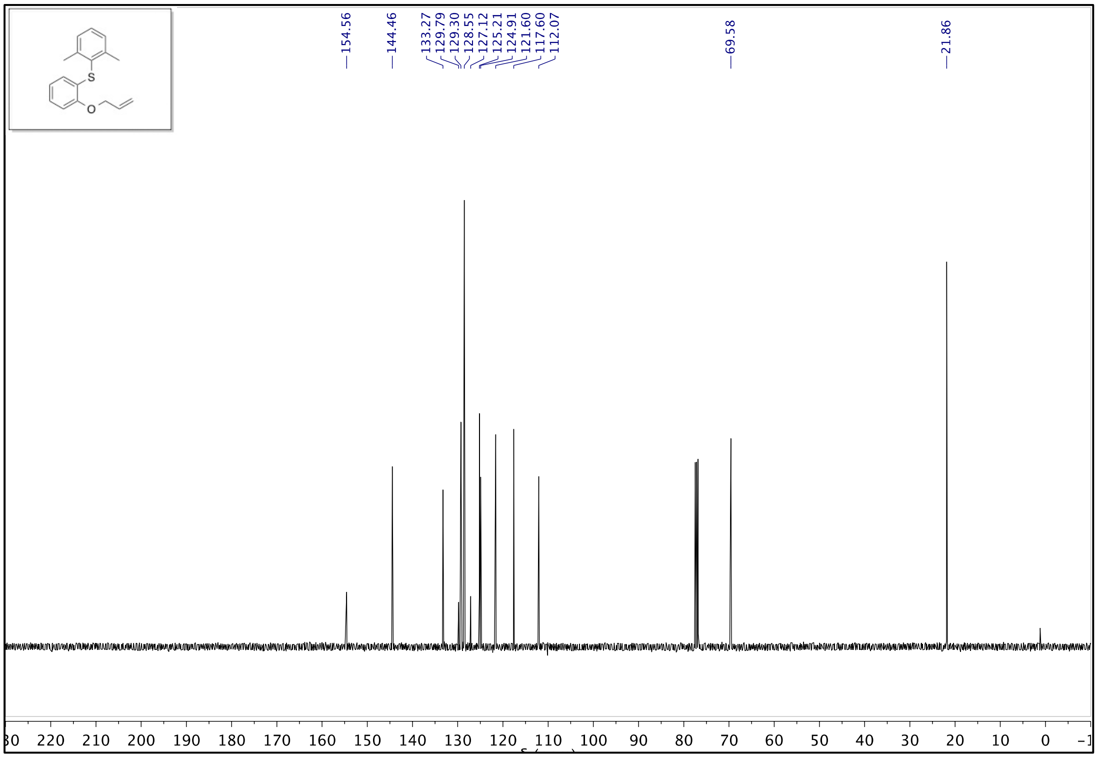

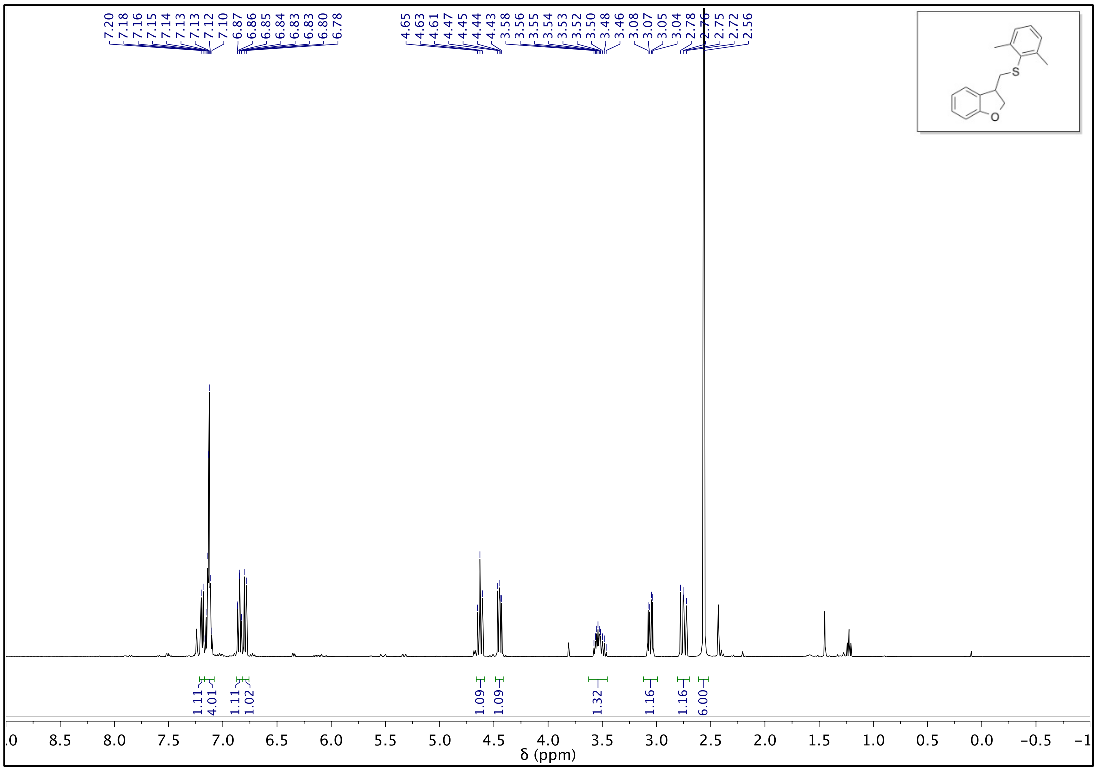
7.20
7.18
7.16
7.15
7.14
7.13
7.13
7.12
7.10
6.87
6.86
6.85
6.84
6.83
6.83
6.80
6.78
4.65
4.63
4.61
4.47
4.45
4.44
4.43
3.58
3.56
3.55
3.54
3.53
3.52
3.50
3.48
3.46
3.08
3.07
3.05
3.04
2.78
2.76
2.75
2.72
2.56
O
S
1.11
4.01
1.11
1.02
1.09
1.09
1.32
1.16
1.16
6.00
δ (ppm)

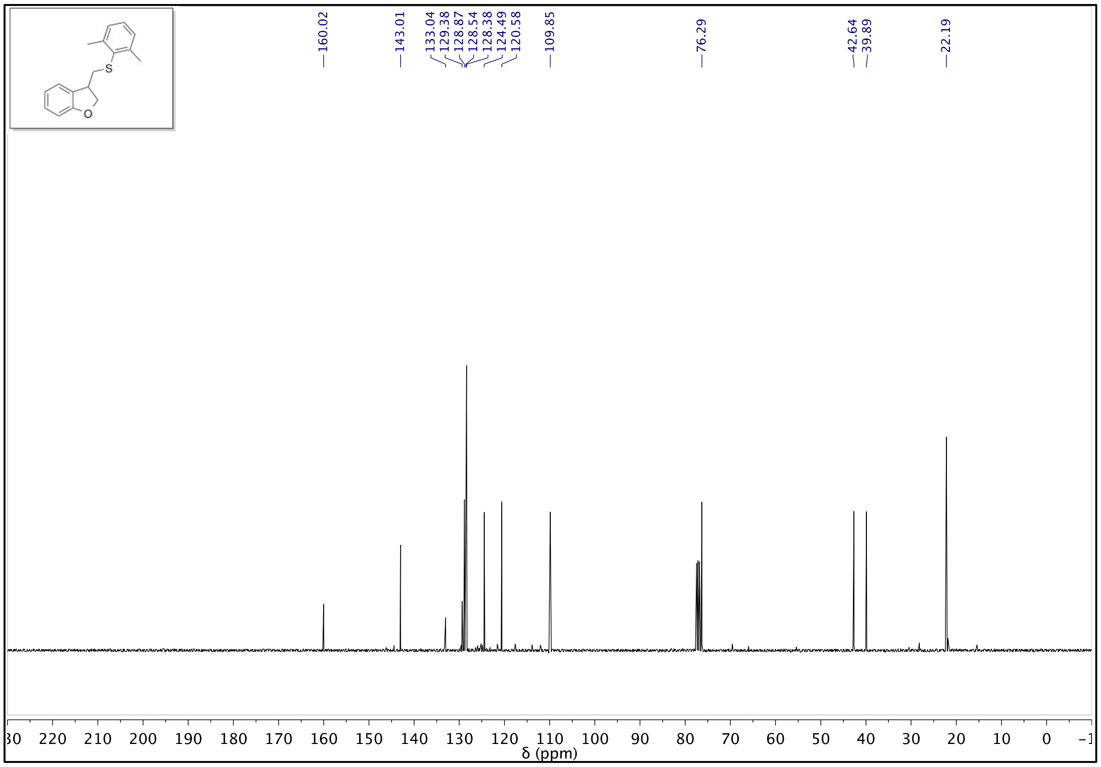
160.02
143.01
133.04
129.38
128.87
128.54
128.38
124.49
120.58
109.85
76.29
42.64
39.89
22.19
S
O
δ (ppm)

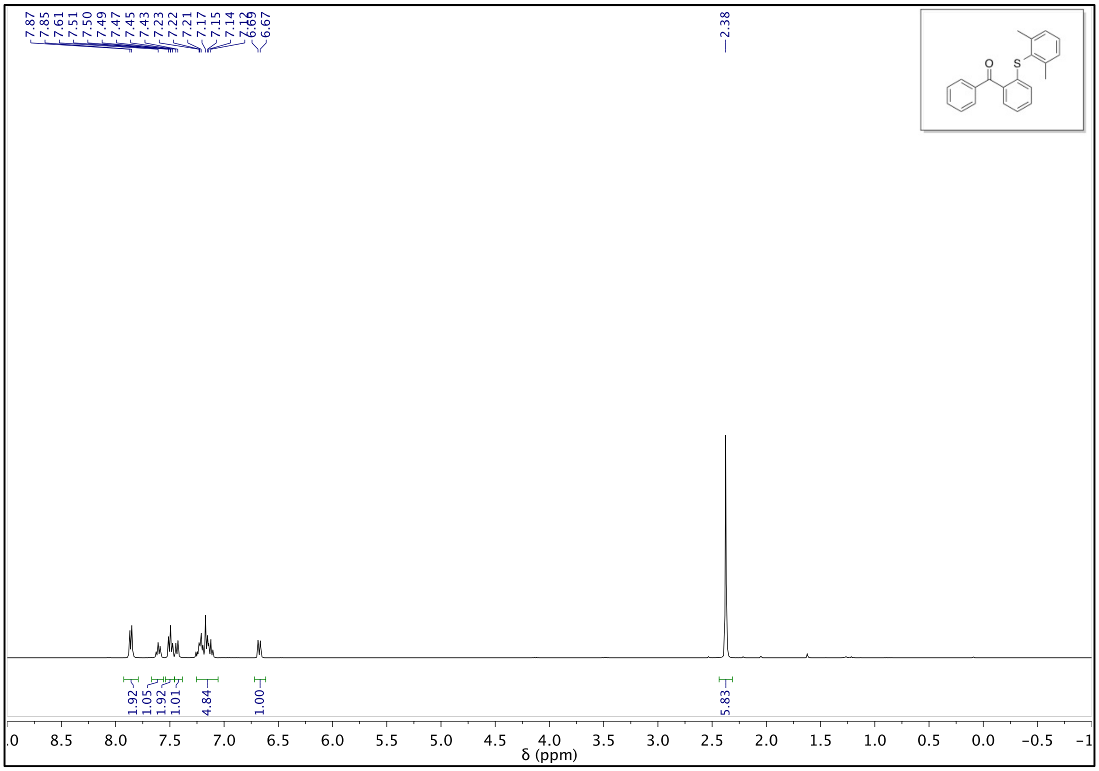
7.87
7.85
7.61
7.51
7.50
7.49
7.47
7.45
7.43
7.23
7.22
7.21
7.17
7.15
7.14
7.12
6.69
6.67
2.38
1.92
1.05
1.92
1.01
4.84
1.00
5.83
δ (ppm)

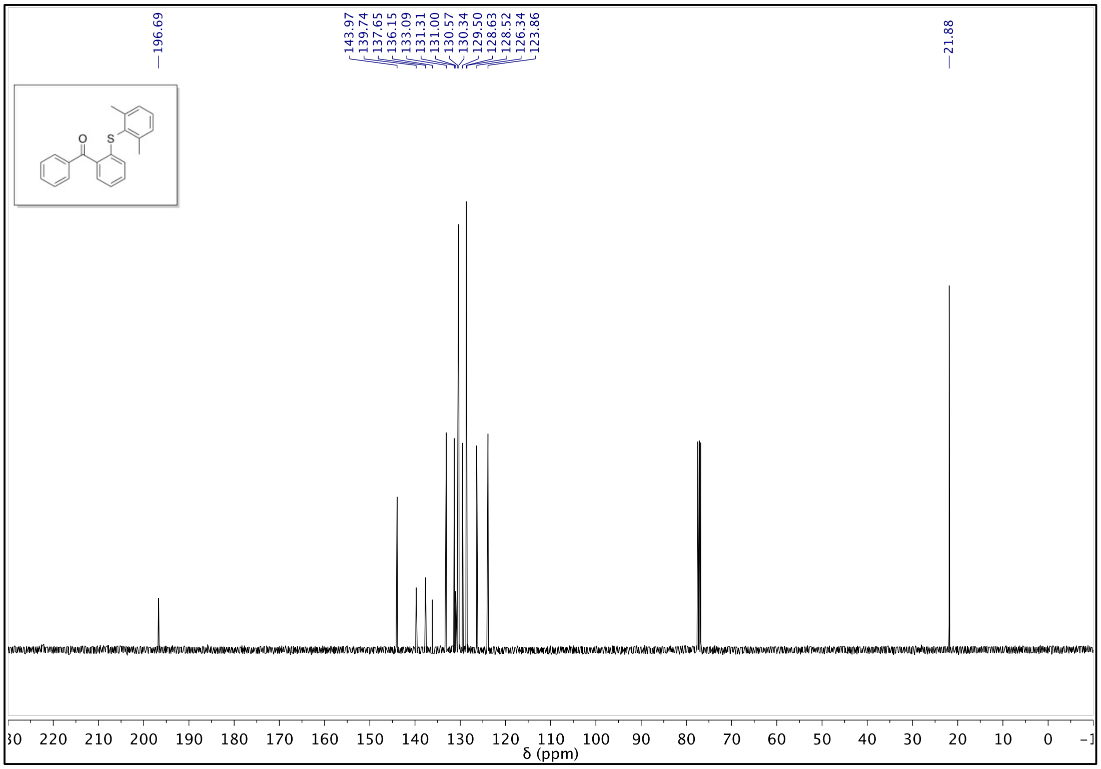
196.69
143.97
139.74
137.65
136.15
133.09
131.31
131.00
130.57
130.34
129.50
128.63
128.52
126.34
123.86
21.88
δ (ppm)

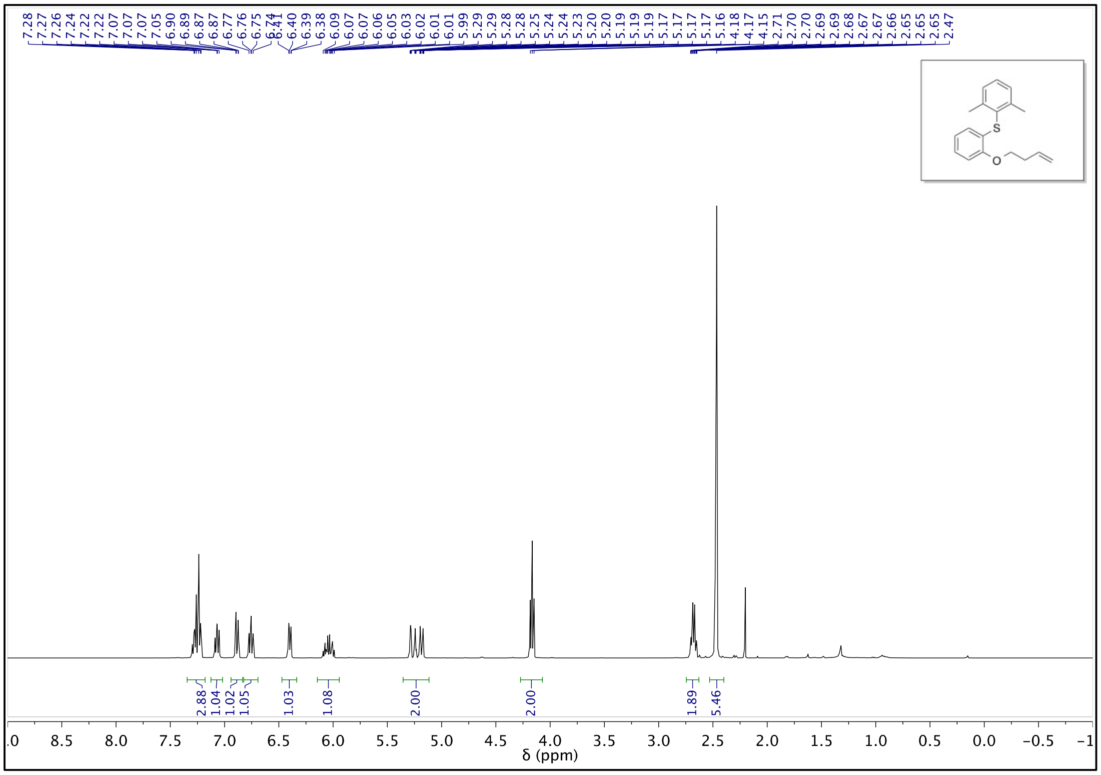

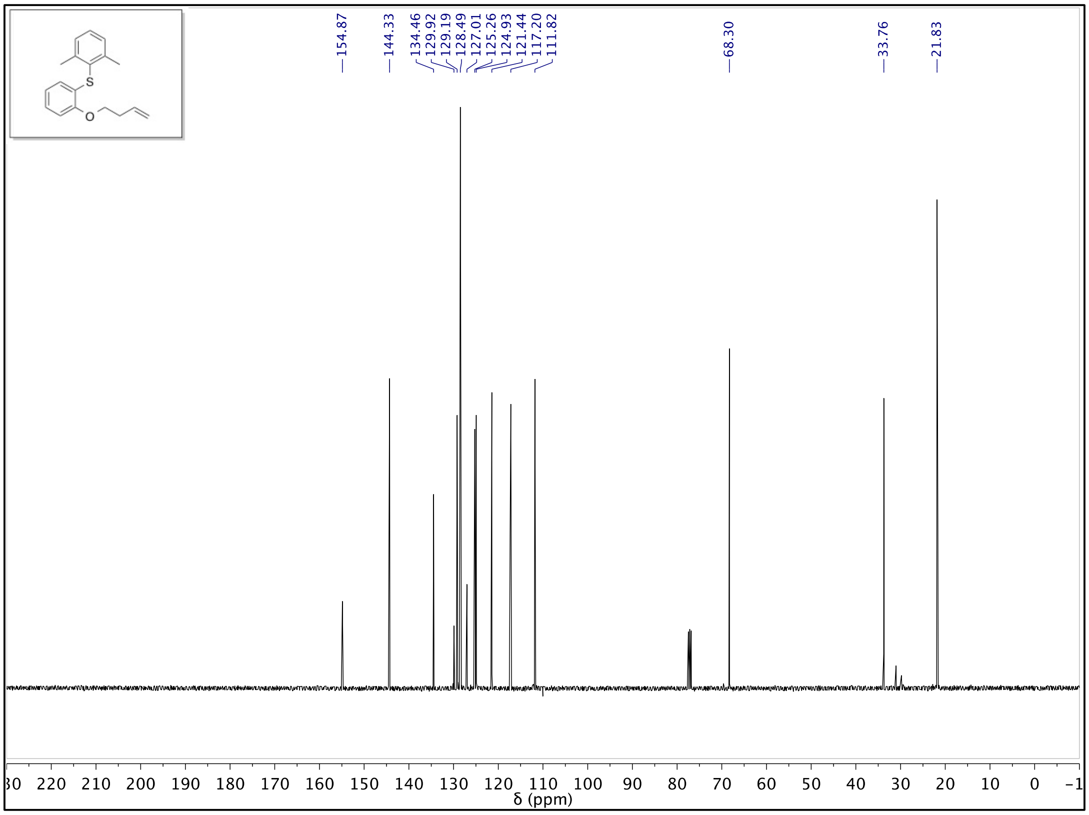

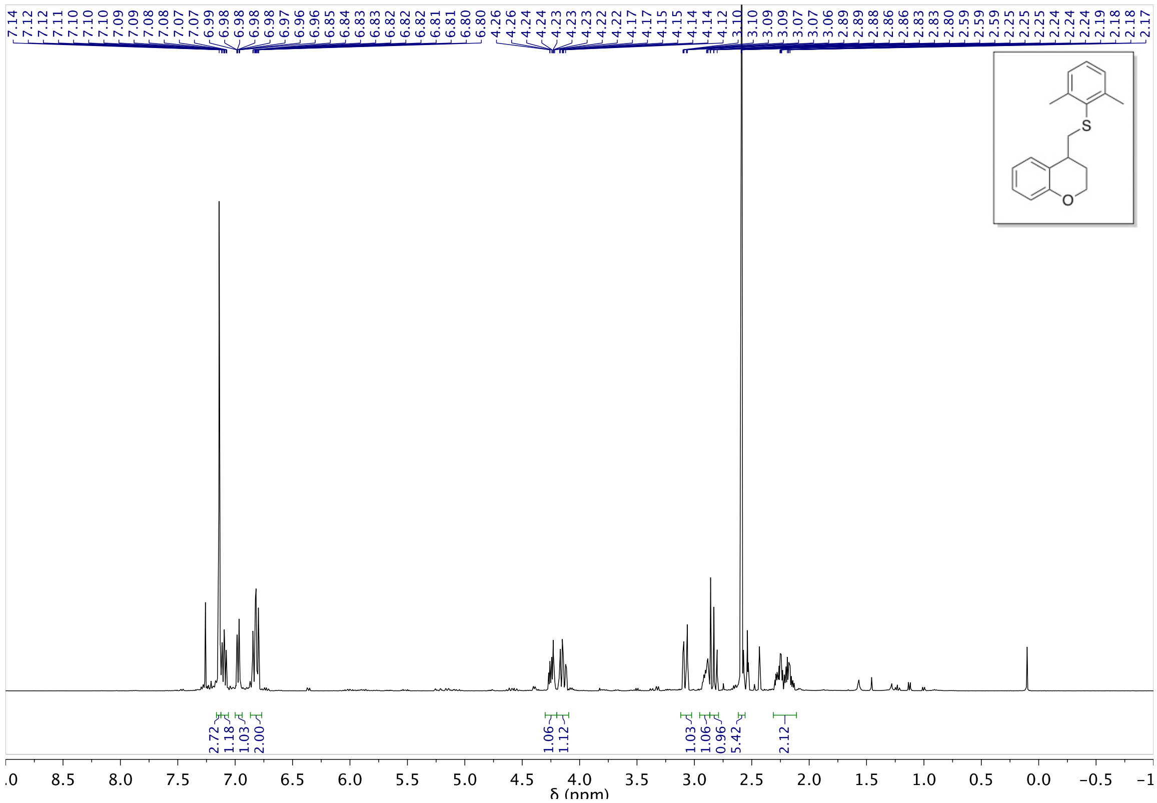

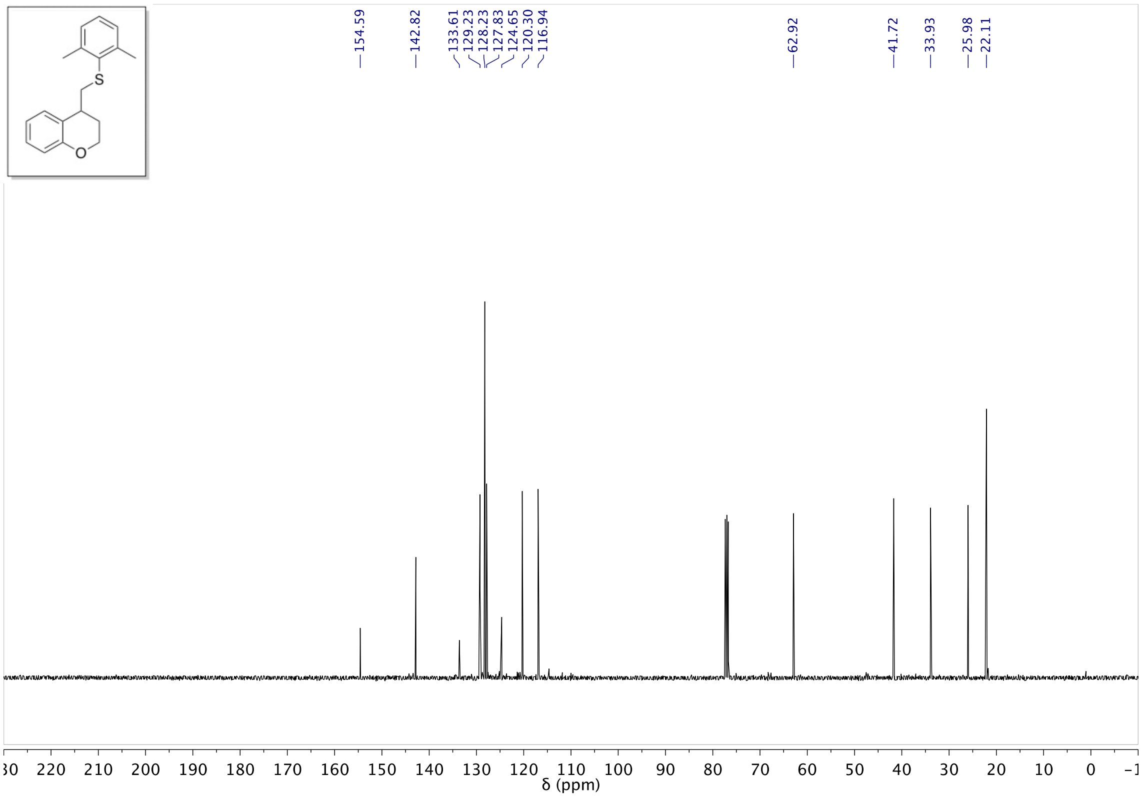

Supplement: SC-007-C5SC04709A-s001 [file SC-007-C5SC04709A-s001.pdf]
